# Supplementary material for: Malnutrition enteropathy in Zambian and Zimbabwean children with severe acute malnutrition: A multi-arm randomized phase II trial
Source: Nat Commun. 2024 Apr 17;15:2910. doi: 10.1038/s41467-024-45528-0 (PMC11024201; doi:10.1038/s41467-024-45528-0)
Supplement: Supplementary file 1 — Supplementary Information [file 41467_2024_45528_MOESM1_ESM.pdf]

Novel therapies for malnutrition enteropathy in children with severe acute malnutrition.
SUPPLEMENTARY APPENDIX

Table of Contents **Page**

Table of Contents	Page
Supplementary Table S1 – Heterogeneity by site	2
Supplementary Table S2 – Effects of transformation on ANCOVA analysis	3
Supplementary Table S3 – Clinical Adverse Events	4
Supplementary Table S4 – Laboratory Adverse Events	5
Supplementary Table S5 – ELISA assay kits used	6
Supplementary Note 1 CONSORT checklist	7
Supplementary Note 2 Trial protocol	10
Supplementary Note 3 Statistical Analysis Plan	108

Supplementary Table S1 Heterogeneity of effects: site-specific analysis of interventions on primary and secondary endpoints, based on non-transformed variables

	Whole group (n=125)	Lusaka only (n=62)	Harare only (n=63)
Primary endpoint	Teduglutide: -0.89 (-1.69,-0.09); P=0.07	n.s.	Teduglutide: -2.08 (-3.48,-0.68); P=0.02<br>Budesonide: -1.38 (-2.70,-0.06); P=0.09
Plasma LBP	n.s.	n.s.	n.s.
Plasma sCD14	n.s.	NAG: -0.0001 (-0.0002,-9x10^{-6}): p=0.07	n.s.
Plasma CD163	Budesonide: -405 (-738,-73); P=0.05	Colostrum: -0.39 (-0.65,-0.13); P=0.02<br>Budesonide: -0.38 (-0.65,-0.10): P=0.03	Teduglutide: -0.45 (-0.77,-0.13); P=0.02
Plasma C-Reactive Protein	Colostrum: -5.9 (-10.3,-1.4); P=0.03<br>NAG: -4.8 (-9.2,-0.5); P=0.07<br>Budesonide: -5.2 (-9.6,-0.8); P=0.05	n.s.	NAG: -1.43 (-2.5,-0.36); P=0.03<br>Teduglutide: -1.37 (-2.43,-0.31); P=0.04<br>Budesonide: -1.18 (-2.21,-0.16); P=0.06
Plasma intestinal fatty acid binding protein (iFABP)	n.s.	NAG: 20.9 (3.0,38.7): P=0.06<br>Teduglutide: 20.3 (3.14,37.5); P=0.05	n.s.
Plasma GLP-2	n.s.	n.s.	n.s.
Change in weight between baseline and day 15	n.s.	n.s.	Colostrum: 0.71 (0.22,1.2): P=0.02<br>Budesonide: 0.66 (0.17,1.14): P=0.03
Change in height between baseline and day 15	n.s.	Teduglutide: 0.63 (0.04,1.2): P=0.08	n.s.
Change in MUAC between baseline and day 15	n.s.	n.s.	n.s.

NAG, N-acetyl glucosamine. n.s. no significant result. Confidence limits are 90% confidence limits and pre-specified significance level -= 0.10. All statistical testing was 2-sided. Site specific effects only shown at that confidence level or less.

Supplementary Table S2 Effects of transformation on ANCOVA analysis of primary and secondary endpoints

	Untransformed, as reported in main paper	Transformation	Intervention effects when transformed
Primary endpoint	Teduglutide: -0.89 (-1.69,-0.10); *P*=0.07	Trichotomisation	n.s.
Plasma LBP	n.s.	Square root	n.s.
Plasma sCD14	n.s.	Inverse square root	n.s.
Plasma CD163	Budesonide: -405 (-738,-73); P=0.05	Logarithmic	Budesonide: -0.246 (-0.452,-0.040); P=0.05
Plasma C-Reactive Protein	Colostrum: -5.9 (-10.3,-1.4); P=0.03<br>NAG: -4.8 (-9.2,-0.5); P=0.07<br>Budesonide: -5.2 (-9.6,-0.8); P=0.05	Logarithmic	Budesonide: -0.758 (-1.509,-0.007); P=0.097
Plasma intestinal fatty acid binding protein (iFABP)	n.s.	Square root	n.s.
Plasma GLP-2	n.s.	Logarithmic	Colostrum: 0.269 (0.008,0.530); P=0.09

NAG, N-acetyl glucosamine. n.s. no significant effects. All statistical testing was 2-sided.

Supplementary Table S3 Clinical adverse events by treatment arm

	Colostrum	NAG	Teduglutide	Budesonide	Standard care
**Unrelated SAEs**					
Total	1	1	4	2	2
Readmission	1	1	3	1	1
Death	0	0	1	1	1
**Unrelated AEs**					
Total	13	17	22	18	22
Fever	4	5	4	6	3
Altered mental status	0	1	1	0	0
Respiratory symptoms	1	3	1	6	4
Tuberculosis	0	0	1	0	0
Diarrhoea	4	2	5	2	5
Vomiting	2	2	3	2	1
Rash	1	0	3	1	1
Worsened oedema	0	0	1	0	2
Eye discharge	0	1	0	0	0
Jaundice	1	0	0	0	0
Injection site soreness, not IMP-related	0	0	1	0	2
Anorexia	0	2	0	0	0
Hypoglycaemia	0	0	1	0	0
Hypothermia	0	1	0	0	0
Malaria (confirmed)	0	0	0	0	1
Oral candidiasis	0	0	0	1	2
Scabies	0	0	0	0	1
Seizure	0	0	1	0	0

Supplementary Table S4 Laboratory adverse events in each treatment arm

A Laboratory abnormalities at baseline

	**Colostrum**	**NAG**	**Teduglutide**	**Budesonide**	**Standard care**
High K	11	8	13	10	9
High ALT	9	10	14	5	10
High AST	11	14	16	14	15
High ALP	0	0	0	1	2
High bilirubin	0	0	1	1	0
High creatinine	1	0	0	0	0
Low Hb	18	14	18	15	22
Low platelets	0	0	1	1	0
Low Na	6	11	9	6	10
Low phosphate	3	2	2	3	3
Low bicarbonate	14	13	15	10	13
Low albumin	21	21	24	21	23
Totals	99	93	113	87	107

B New laboratory abnormalities on day 5

	**Colostrum**	**NAG**	**Teduglutide**	**Budesonide**	**Standard care**
High K	4	5	4	6	4
High ALT	1	1	2	5	4
High AST	5	2	2	5	3
High ALP	1	0	0	0	0
High creatinine	1	0	0	1	1
Low Hb	5	9	5	4	1
Low Na	2	2	3	7	3
Low K	1	1	0	0	0
Low phosphate	0	2	0	0	0
Low bicarbonate	5	6	7	9	8
Low albumin	0	1	1	0	4
Totals	25	29	24	37	28

C New laboratory abnormalities on day 15

	**Colostrum**	**NAG**	**Teduglutide**	**Budesonide**	**Standard care**
High K	6	3	4	4	2
High ALT	4	3	3	1	1
High AST	2	4	7	3	2
High ALP	0	0	1	0	0
Low Hb	3	0	2	0	1
Low Na	6	5	1	7	2
Low bicarbonate	7	7	3	6	1
Low albumin	0	0	1	0	1
Totals	28	22	22	21	10

Supplementary Table S5 ELISA assays used

Biomarker	Specimen type	TAME	Company
A1AT	Stool	Immuchrom; Cat # IC6200	Oxford Biosystems
CRP	Blood	R&D Systems, Quantikine ELISA Cat # DCRP00	Biotechne
GLP-2	Blood	EMD Millipore Corporation ELISA kit, Cat # EZGLP2 – 37K	Merck
I-FABP	Blood	Hycult Biotech HK 406-2	Cambridge Bioscience
IGF-1	Blood	R&D Systems, DuoSet ELISA Cat # SG100B	Biotechne
LBP	Blood	R&D Systems, DuoSet ELISA Cat # DY870-05	Biotechne
MPO	Stool	ImmunDiagnostik AG, ELISA Kit K6630	Biohit healthcare
NEO	Stool	Arigobio ARG80878	Arigo Biolaboratories
sCD14	Blood	R&D Systems, Quantikine ELISA; Cat # DC140	Biotechne
sCD163	Blood	R&D Systems, Quantikine ELISA Cat # DC1630	Biotechne

Supplementary Note 1 CONSORT checklist

CONSORT 2010 checklist of information to include when reporting a randomised trial*

Section/Topic	Item No	Checklist item	Reported on page No
**Title and abstract**			
	1a	Identification as a randomised trial in the title	1
	1b	Structured summary of trial design, methods, results, and conclusions (for specific guidance see CONSORT for abstracts)	2
**Introduction**			
Background and objectives	2a	Scientific background and explanation of rationale	3
	2b	Specific objectives or hypotheses	3
**Methods**			
Trial design	3a	Description of trial design (such as parallel, factorial) including allocation ratio	10
	3b	Important changes to methods after trial commencement (such as eligibility criteria), with reasons	12
Participants	4a	Eligibility criteria for participants	9
	4b	Settings and locations where the data were collected	4
Interventions	5	The interventions for each group with sufficient details to allow replication, including how and when they were actually administered	10-11
Outcomes	6a	Completely defined pre-specified primary and secondary outcome measures, including how and when they were assessed	11-12
	6b	Any changes to trial outcomes after the trial commenced, with reasons	12
Sample size	7a	How sample size was determined	12
	7b	When applicable, explanation of any interim analyses and stopping guidelines	N/A
Randomisation:			
Sequence generation	8a	Method used to generate the random allocation sequence	10
	8b	Type of randomisation; details of any restriction (such as blocking and block size)	10

Allocation concealment mechanism	9	Mechanism used to implement the random allocation sequence (such as sequentially numbered containers), describing any steps taken to conceal the sequence until interventions were assigned	10
Implementation	10	Who generated the random allocation sequence, who enrolled participants, and who assigned participants to interventions	10
Blinding	11a	If done, who was blinded after assignment to interventions (for example, participants, care providers, those assessing outcomes) and how	11
	11b	If relevant, description of the similarity of interventions	N/A
Statistical methods	12a	Statistical methods used to compare groups for primary and secondary outcomes	13
	12b	Methods for additional analyses, such as subgroup analyses and adjusted analyses	13
**Results**			
Participant flow (a diagram is strongly recommended)	13a	For each group, the numbers of participants who were randomly assigned, received intended treatment, and were analysed for the primary outcome	4, and Fig 1
	13b	For each group, losses and exclusions after randomisation, together with reasons	4, and Fig 1
Recruitment	14a	Dates defining the periods of recruitment and follow-up	4
	14b	Why the trial ended or was stopped	12
Baseline data	15	A table showing baseline demographic and clinical characteristics for each group	Table 1
Numbers analysed	16	For each group, number of participants (denominator) included in each analysis and whether the analysis was by original assigned groups	Figure 1
Outcomes and estimation	17a	For each primary and secondary outcome, results for each group, and the estimated effect size and its precision (such as 95% confidence interval)	5-6, and Tables 3 and 4
	17b	For binary outcomes, presentation of both absolute and relative effect sizes is recommended	N/A, except for mortality but there were too few deaths for statistical analysis
Ancillary analyses	18	Results of any other analyses performed, including subgroup analyses and adjusted analyses, distinguishing pre-specified from exploratory	4-5, and supplementary table S1
Harms	19	All important harms or unintended effects in each group (for specific guidance see CONSORT for harms)	6, and supplementary

			tables S3 and S4
**Discussion**			
Limitations	20	Trial limitations, addressing sources of potential bias, imprecision, and, if relevant, multiplicity of analyses	8
Generalisability	21	Generalisability (external validity, applicability) of the trial findings	8-9
Interpretation	22	Interpretation consistent with results, balancing benefits and harms, and considering other relevant evidence	6-9
**Other information**			
Registration	23	Registration number and name of trial registry	9
Protocol	24	Where the full trial protocol can be accessed, if available	Protocol is included in the Supplementary Information (see below)
Funding	25	Sources of funding and other support (such as supply of drugs), role of funders	21

Citation: Schulz KF, Altman DG, Moher D, for the CONSORT Group. CONSORT 2010 Statement: updated guidelines for reporting parallel group randomised trials. BMC Medicine. 2010;8:18.
© 2010 Schulz et al. This is an Open Access article distributed under the terms of the Creative Commons Attribution License (http://creativecommons.org/licenses/by/2.0), which permits unrestricted use, distribution, and reproduction in any medium, provided the original work is properly cited.

*We strongly recommend reading this statement in conjunction with the CONSORT 2010 Explanation and Elaboration for important clarifications on all the items. If relevant, we also recommend reading CONSORT extensions for cluster randomised trials, non-inferiority and equivalence trials, non-pharmacological treatments, herbal interventions, and pragmatic trials. Additional extensions are forthcoming: for those and for up-to-date references relevant to this checklist, see www.consort-statement.org.

1.0 Title Page

Full / Long Title

Therapeutic approaches to malnutrition enteropathy: phase II trials of four novel interventions in children in Zambia and Zimbabwe

Short title and/or Acronym

Therapeutic Approaches to Malnutrition Enteropathy (TAME)

Sponsor

Queen Mary University of London

Representative of the Sponsor:

Dr Mays Jawad

Research & Development Operations Manager

Joint Research Management Office

QM Innovation Building

5 Walden Street

London

E1 2EF

Phone: 020 7882 7260

Email: sponsorsrep@bartshealth.nhs.uk

Sponsor Reference 011724 QM

2.0 Research Reference Numbers

UNZABREC (Zambia) Number: 006-09-17

MRCZ (Zimbabwe) Number: MRCZ/A/2458

ReDA Number :	011724 QM
ISRCTN:	NCT03716115
ZAMRA (Zambia) Number:	CT 082/ 18
MCAZ (Zimbabwe) Number:	CT/176/2019
JREC (Zimbabwe) Number:	JREC/66/19

3.0 Signature Pages

Chief Investigator Declaration

I confirm that the following protocol (version 2.0, dated 1st November 2019), has been written by me and I, as the Chief Investigator, agree to conduct the trial in compliance with this version of the protocol.

I will adhere to the principles outlined in the Medicines for Human Use (Clinical Trials) Regulations 2004 (SI 2004/1031), and all subsequent amendments of the clinical trial regulations, current Research Governance Framework, the World Medical Association Declaration of Helsinki (1996), GCP guidelines, the Sponsor's SOPs, and other regulatory requirements as amended. I will adhere to legislation in force in Zambia: The National Health research Act 2013 and the Medicine and Allied Substances Act 2013. I will also adhere to legislation in force in Zimbabwe: the Medicines and Allied Substances Control Act and guidelines for GCP 2012 Zimbabwe.

I agree to ensure that the confidential information contained in this document will not be used for any other purpose other than the evaluation or conduct of the clinical investigation without the prior written consent of the sponsor.

I also confirm that I will make the findings of the study publicly available through publication and/or other dissemination tools without any unnecessary delay and that an honest accurate and transparent account of the study will be given; and that any discrepancies from the study as planned in this protocol will be explained.

Chief Investigator: Professor Paul Kelly

Chief Investigator Site: University of Zambia School of Medicine and Queen Mary University of London

Signature: ....

Date: ....../....../......

Name (please print):

....

Statistician Declaration

The clinical study as detailed within this research protocol (version 2.0, dated 1st November 2019) involves the use of an investigational medicinal product and will be conducted in accordance with the current Research Governance Framework for Health & Social Care, the World Medical Association Declaration of Helsinki (1996), Principles of ICH E6-GCP, ICH E9 -Statistical principles for Clinical Trials, ICH E10 - Choice of Control Groups and the current regulatory requirements, as detailed in the Medicines for Human Use (Clinical Trials) Regulations 2004 (UK S.I. 2004/1031) and any subsequent amendments of the clinical trial regulations.

Statistician: Dr Kelley Van Buskirk

Job title: Statistician

Statistician Site/Organisation: 4401 Arlington avenue Fort Wayne, IN 46807, USA

Signature: ....

Date: ....../....../......

Name (please print): ....

Principal Investigator: Zambia

I, as Principal Investigator confirm that I have read and understood the following protocol (version 2.0, dated 1st November 2019). I agree to conduct the trial in compliance with this version of the protocol. I will adhere to the principles outlined in the Medicines for Human Use (Clinical Trials) Regulations 2004 (SI 2004/1031), and any subsequent amendments of the clinical trial regulations, current Research Governance Framework, GCP guidelines, the World Medical Association Declaration of Helsinki (1996), the Sponsor's SOPs, and other regulatory requirements as amended. I will adhere to legislation in force in Zambia: The National Health research Act 2013 and the Medicine and Allied Substances Act 2013.

I agree to ensure that the confidential information contained in this document will not be used for any other purpose other than the evaluation or conduct of the clinical investigation without the prior written consent of the sponsor.

Principal Investigator Name: Dr Beatrice Amadi

Principal Investigator Site: University Teaching Hospital, Lusaka, Zambia.

Signature:..

Date: ....../....../......

Name (please print): ....

This page must be signed by each PI at every site, and kept in the ISF, a copy of this page must be sent to the lead site/coordinating centre as evidence.

Principal Investigator: Zimbabwe

I, as Principal Investigator confirm that I have read and understood the following protocol (version 2.0, dated 1st November 2019). I agree to conduct the trial in compliance with this version of the protocol. I will adhere to the principles outlined in the Medicines for Human Use (Clinical Trials) Regulations 2004 (SI 2004/1031), and any subsequent amendments of the clinical trial regulations, current Research Governance Framework, GCP guidelines, the World Medical Association Declaration of Helsinki (1996), the Sponsor's SOPs, and other regulatory requirements as amended. I will adhere to legislation in force in Zimbabwe: the Medicines and Allied Substances Control Act and guidelines for GCP 2012 Zimbabwe.

I agree to ensure that the confidential information contained in this document will not be used for any other purpose other than the evaluation or conduct of the clinical investigation without the prior written consent of the sponsor.

Principal Investigator Name: Dr Mutsa Bwakura-Dangarembizi

Principal Investigator Site: Parirenyatwa Hospital, Harare, Zimbabwe

Signature:..

Date: ....../....../......

Name (please print): ....

This page must be signed by each PI at every site, and kept in the ISF, a copy of this page must be sent to the lead site/coordinating centre as evidence.

Principal Investigator: Zvitambo

I, as Principal Investigator confirm that I have read and understood the following protocol (version 2.0, dated 1st November 2019). I agree to conduct the trial in compliance with this version of the protocol. I will adhere to the principles outlined in the Medicines for Human Use (Clinical Trials) Regulations 2004 (SI 2004/1031), and any subsequent amendments of the clinical trial regulations, current Research Governance Framework, GCP guidelines, the World Medical Association Declaration of Helsinki (1996), the Sponsor's SOPs, and other regulatory requirements as amended. I will adhere to legislation in force in Zimbabwe: the Medicines and Allied Substances Control Act and guidelines for GCP 2012 Zimbabwe.

I agree to ensure that the confidential information contained in this document will not be used for any other purpose other than the evaluation or conduct of the clinical investigation without the prior written consent of the sponsor.

Principal Investigator Name: Professor Andrew Prendergast

Principal Investigator Site: Zvitambo Institute for Maternal and Child Health Research, Harare, Zimbabwe

Signature:..

Date: ....../....../......

Name (please print): ....

This page must be signed by each PI at every site, and kept in the ISF, a copy of this page must be sent to the lead site/coordinating centre as evidence.

4.0 Key Trial Contacts

Chief Investigator	Professor Paul Kelly MD FRCP<br>TROPGAN<br>UNZA School of Medicine<br>University Teaching Hospital<br>Lusaka, Zambia<br>*and*<br>Blizard Institute<br>Queen Mary University of London<br>4 Newark Street<br>London E1 2AT, UK<br>email: m.p.kelly@qmul.ac.uk<br>tel: +44 (0) 20 7882 2643<br>and: +260 966 751875
Trial Co-ordinators/Managers	Ms Miyoba Chipunza (Zambia)<br>Clinical Research Coordinator<br>TROPGAN<br>UNZA School of Medicine<br>University Teaching Hospital<br>Lusaka, Zambia.<br>Lusaka<br>email: miyoba@tropgan.com<br>tel: +260 965 327070<br><br>Mrs Virginia Sauramba (Zimbabwe)<br>Research Administration, Compliance Manager and Executive Assistant to the Director<br>Zvitambo Institute For Maternal And Child Health Research<br>No. 16 Lauchlan Ave., Meyrick Park, Mabelreign

	Harare, Zimbabwe<br>Tel: +263-4-306028, 306259, 306056<br>Fax: +263-4-311069<br>Cell: +263 783 910 622, +263 712 620 783<br>E-mail: vsauramba@zvitambo.co.zw<br>vsauramba@gmail.com
Sponsor	Dr Mays Jawad<br>**Director of Research Services and Business Development**<br>**Queen Mary University of London**<br>Joint Research Management Office (JRMO)<br>Queen Mary Innovation Centre<br>Lower Ground Floor<br>5 Walden Street<br>London, E1 2EF<br>**Tel: 020 7882 7275**<br>Office fax: **020 7882 7276**<br>E-mail: researchgovernance@qmul.ac.uk
Laboratories	1 Tropical Gastroenterology and Nutrition Group (TROPGAN), University of Zambia School of Medicine, Lusaka, Zambia<br>2 Zvitambo Institute for Maternal and Child Health Research, 16 Lauchlan Avenue, Meyrick Park, Harare Zimbabwe .<br>3 Blizard Institute, Queen Mary University of London, London, UK
Funder(s)	Medical Research Council<br>2nd Floor, David Phillips Building,<br>Polaris House<br>North Star Avenue<br>Swindon SN2 1FL

	**Email:** Lucy.Strange@headoffice.mrc.ac.uk<br>**Web:** www.mrc.ac.uk<br><br>The Wellcome Trust<br>205 Euston Road<br>London NW1 2BE<br>**Web**: www.wellcome.ac.uk
Clinical Trials Unit	n/a
Statistician	Dr Kelley van Buskirk,<br>4401 Arlington Avenue,<br>Fort Wayne, IN 46807<br>USA<br>email: kelley.vanbuskirk@gmail.com<br>tel: +1 260-387-6190
Trial pharmacist	Rizvan Batha<br>Head of Barts Health Pharmaceuticals<br>Assistant Chief Pharmacist<br>Barts Health NHS Trust<br>The Royal London Hospital<br>Pathology & Pharmacy Building<br>80 Newark Street, London E1 2ES<br>tel: +44 7703 469468<br>email: rizvan.batha@nhs.net<br><br>Jimmy Hangoma<br>UNZA School of Medicine<br>University Teaching Hospital<br>Lusaka, Zambia.<br>TROPGAN<br>Lusaka

	Email: jimmyhangoma0282@gmail.com<br>tel: +260 977534813<br><br>Shepherd Mudzingwa<br>University of Zimbabwe Clinical Research Centre<br>2 Allan Wilson<br>Belgravia, Harare<br>Zimbabwe.<br>Email: smudzingwa@uzcrc.org
Committees<br>(DMEC, TSC, TMG)	*Trial Management Group:*<br>Professor Paul Kelly (chair)<br>Professor Andrew Prendergast<br>Dr Beatrice Amadi<br>Dr Mutsa Bwakura-Dangarembizi<br>Dr Kanta Chandwe<br>Dr Susan Hill<br>Professor Simon Murch<br>Professor Ray Playford<br>Dr Jonathan Sturgeon<br><br>*Trial Steering Committee:*<br>Prof James Berkley (chair)<br>Prof Ian Sanderson<br>Prof James Wason (statistician)<br><br>*DMEC:*<br>Professsor Jim Todd (chair)<br>Prof Rose Kambarami<br>Dr Veronica Mulenga<br>Dr Philip Ayieko (statistician)

5.0 Trial Summary

Full title	Therapeutic approaches to malnutrition enteropathy: phase II trials of four novel interventions in children in Zambia and Zimbabwe
Short title and/or Acronym	Therapeutic approaches to malnutrition enteropathy (TAME) trial
Trial Design Methodology	Phase II clinical trial in children, with multi-arm design
Phase of the Trial	II
Study Duration	24 months
Study setting	Multi-site international trial in three centres:<br><br>1 University Teaching Hospital, Lusaka, Zambia<br>2 Parirenyatwa Hospital, Harare, Zimbabwe<br>3 Harare Central Hospital, Harare, Zimbabwe
Investigational Medicinal Product(s)	1 Teduglutide (Takeda)<br>2 Budesonide (Alliance)
Nutritional Products	1 Colostrum (bovine, Colostrum UK)<br>2 N-Acetyl Glucosamine (Blackburn Distributors)
Medical condition or disease under investigation	Severe Acute Malnutrition (SAM) in African children
Planned Sample Size	125 children with SAM
(Maximum) Treatment duration	14 days
Follow up duration	28 days after completion of treatment (28-42 days window)

End of Trial definition	Completion of follow up of the last participant and primary outcome laboratory assays

6.0 Protocol Contributors

Key Protocol Contributors	Full contact details including phone, email and fax numbers
Professor Paul Kelly	Professor Paul Kelly MD FRCP<br>TROPGAN,<br>UNZA School of Medicine, University Teaching Hospital<br>Lusaka, Zambia<br>and<br>Blizard Institute, Queen Mary University of London<br>4 Newark Street, London E1 2AT, UK<br>email: m.p.kelly@qmul.ac.uk<br>tel: +44 (0) 20 7882 2643<br>and: +260 966 751875
Dr Andrew Prendergast	Professor Andrew Prendergast MA DPhil MRCPCH<br>Zvitambo Institute for Maternal and Child Health Research.<br>16 Lauchlan Avenue<br>Meyrick Park, Harare, Zimbabwe<br>and<br>Blizard Institute,<br>Queen Mary University of London,<br>4 Newark Street,<br>London E1 2AT,UK.<br>email: a.prendergast@qmul.ac.uk<br>tel: +263 4 306028<br>and +44 207 882 2269
Dr Beatrice Amadi	Dr Beatrice Amadi MD MMed DipPaedGastroenterology<br>Department of Paediatrics and Child Health<br>University Teaching Hospital<br>Lusaka, Zambia<br>email: beatriceamadi@ymail.com<br>tel: +260 966 752739

Dr Mutsa Bwakura-Dangarembizi	Dr Mutsa Bwakura-Dangarembizi MMed, MSc (Clin Epi)<br>Department of Paediatrics and Child Health,<br>University of Zimbabwe College of Health Sciences,<br>.Harare, Zimbabwe<br>email: mbwakura@medsch.uz.ac.zw<br>tel: +263 2424 791631<br>+263 772601735
The Sponsor has overseen the design of the trial, which has been completed by the research team, and has given valuable advice as to compliance with relevant regulations. The funding agency has had no role in the design of the trial and will not control any aspect of the trial, including design, conduct, data analysis and interpretation, manuscript writing, or dissemination of results.	

7.0 List of contents

1.0 Title Page 1

2.0 Research Reference Numbers 2

3.0 Signature Pages 3

4.0 Key Trial Contacts 8

5.0 Trial Summary 11

6.0 Protocol Contributors 12

7.0 List of Contents 12

8.0 List of Abbreviations / Glossary of Terms 18

9.0 Introduction 20

9.1 Background 20

9.2 Assessment and management 22

9.3 Rationale for study design 23

10.0 Trial Flowchart 24

11.0 Trial Objectives and Design 24

11.1 Primary Objective/s 24

11.2 Secondary Objective/s 24

11.3 Endpoints 24

11.3.1 Primary Endpoint 24

11.3.2 Secondary Endpoints 25

11.4 Exploratory or Tertiary endpoints/outcomes 26

11.5 Objectives and End Points Summary 27

11.6 Trial Design 28
11.7 Study Setting 28
12.0 Eligibility Criteria 28
12.1 Inclusion Criteria 28
12.2 Exclusion Criteria 28
13.0 Trial Procedures 29
13.1 Recruitment 29
13.2 Participant identification 29
13.3 Informed Consent Procedures 29
13.3.1 Responsibility for obtaining consent 29
13.3.2 Consent Considerations 29
13.3.3 Population 29
13.3.4 Vulnerable participant's considerations 30
13.3.5 Written/ reading / translation considerations 30
13.3.6 Participants lacking capacity 30
13.3.7 Minors 30
13.3.8 Consenting process 30
13.3.9 Additional consent provisions for collection and use of participant data and biological specimens in ancillary studies 31
13.4 Screening Procedures 31
13.5 Patient Allocation 31
13.5.1 Randomisation Method 31

13.5.2 Randomisation Procedures 31
13.5.3 Cohort allocation/sequential allocation 31
13.6 Blinding 31
13.7 Unblinding 32
13.8 Trial Schedule 32
13.8.1 Schedule of Treatment for each visit 32
13.8.2 Schedule of Assessment (in Diagrammatic Format) 33
13.8.3 Trial assessments 33
13.8.4 Follow up Procedures 35
13.8.5 Qualitative assessments – Nested studies 35
13.8.6 Radiology Assessments 35
13.9 Withdrawal criteria 35
13.10 Early withdrawal 35
13.11 End of trial (EOT) 35
14.0 Laboratories and samples 36
14.1 Central Laboratories 36
14.2 Local Laboratories 36
14.3 Sample Collection/Labelling/Logging 36
14.4 Sample Receipt/Chain of Custody/Accountability 37
14.5 Sample Analysis Procedures 37
14.5.1 The arrangements for sample analysis 37
14.5.2 Sample Storage Procedures 37

14.6 Sample and Data Recording/Reporting 37
14.7 End of study 37
14.8 Transportation of samples internationally 37
15.0 Trial Interventions 37
15.1 Name and description of investigational medicinal product(s) 39
15.2 Legal status of the drug 39
15.3 Summary of Product Characteristics (SmPC) or IB 39
15.4 Drug storage and supply 39
15.5 Supplier 39
15.6 Manufacturer 39
15.7 How the product should be stored 40
15.8 Details of accountability 40
15.9 Destruction/return and Recall 40
15.10 Prescription of IMP / Placebo/NIMP 40
15.11 Preparation and labelling of IMP and nutritional products 40
15.12 Preparation and Administration of IMP and nutritional products 41
15.13 Dosage schedules 41
15.14 Dispensing of IMP 41
15.15 Dosage modifications 41
15.16 Known drug reactions and interaction with other therapies 42
15.17 Prior and Concomitant medication 42
15.18 Trial restrictions 42

15.19 Assessment of compliance 42
15.20 Name and description of each Non-Investigational Medicinal Product 42
15.21 Arrangements for post-trial access to IMP and care 42
16 Equipment and Devices 43
17 Pharmacovigilance 44
17.1 General Definitions 44
17.2 Site Investigators Assessment 45
17.3 Reference Safety information 46
17.4 Notification and reporting Adverse Events or Reactions 46
17.5 Notification of AEs of special interest 46
17.6 Adverse events that do not require reporting 47
17.7 Notification and Reporting of Serious Adverse Events & SUSARs 47
17.8 Sponsor Medical Assessment 47
17.9 Urgent Safety Measures 47
17.10 Procedures for reporting blinded SUSARs 47
17.11 Pregnancy 47
18.0 Annual reporting 48
19.0 Statistical and Data Analysis 49
19. 1 Sample size calculation 49
19.2 Planned recruitment rate 49
19.3 Statistical analysis plan (SAP) 49
19.4 Summary of baseline data and flow of patients 49

19.5 Primary outcome analysis 50
19.6 Secondary outcome analysis 50
19.7 Subgroup analyses 50
19.8 Adjusted analysis 50
19.9 Interim analysis and criteria for the premature termination of the trial 50
19.10 Subject population 50
19.11 Procedure(s) to account for missing or spurious data 51
19.12 Other statistical considerations. 51
19.13 Economic evaluation 51
20.0 Data Handling & Record Keeping 52
20.1 Confidentiality 52
20.2 Data Custodian Details 52
20.3 Pseudononymisation 52
20.4 Transferring/Transporting Data 52
20.5 Data collection tools and source document identification 52
20.6 Source Data 53
20.7 Case Report Form 53
20.8 CRFs as Source Documents 53
20.9 Data handling and record keeping 53
20.10 Access to Data, Source Data and Documents 54
21.0 Archiving 54
22.0 Monitoring, Audit and Inspection 54

22.1 Monitoring 54
22.2 Auditing 54
22.3 Notification of Serious Breaches to GCP and/or the protocol 54
22.4 Compliance 54
22.5 Non-Compliance 54
22.6 Regulatory Compliance 55
23.0 Financial and other competing interests for the chief investigator,
PIs at each site and committee members for the overall trial management 55
24.0 Ethical and Regulatory Considerations 56
25.0 Peer review 56
26.0 Public and Participant Involvement 56
27.0 Indemnity 56
27.1 Amendments 56
27.2 Access to the final trial dataset 56
28.0 Trial Committees 57
29.0 Publication and Dissemination Policy 57
29.1 Publication 57
29.2 Dissemination policy 57
30.0 References 58
31.0 Appendices/Protocol Amendments 60

8.0 List of Abbreviations / Glossary of Terms

AE	Adverse Event
AR	Adverse Reaction
ART	Anti-Retroviral Therapy
ASR	Annual Safety Report
APR	Annual Progress Report
BMI	Body Mass Index
CD	Cluster of Differentiation
CI	Chief Investigator
CLSM	Confocal Laser Scanning Microscope
CRF	Case Report Form
DSMB	Data & Safety Monitoring Board
EE	Environmental Enteropathy
ELISA	Enzyme-Linked Immunoabsorbent Assay
FABP	Intestinal fatty acid binding protein (also known as I-FABP)
FFPE	Formalin Fixed, Paraffin Embedded
GBP	Great British Pound (currency)
GCP	Good Clinical Practice
GI	Gastrointestinal
GLP	Good laboratory practice
GLP2	Glucagon-Like Peptide 2

HAZ/LAZ	Height for Age Z score (length, recumbent, if under 2 years of age)
HIV	Human Immunodeficiency Virus
HPLC	High Performance Liquid Chromatography
IATA	International Air Transport Association
IBD	Inflammatory Bowel Disease
ICF	Informed Consent Form
IMP	Investigational Medicinal Product
LAL	Limulus Amoebacyte Lysate
LPS	Lipopolysaccharide
LBP	LPS Binding Protein
JRMO	Joint Research Management Office
MMP	Matrix Metallo-Protease
MT	Microbial Translocation
MUAC	Mid-Upper Arm Circumference
MRCZ	Medical Research Committee of Zimbabwe
NIMP	Non Investigational Medicinal Product.
NSAID	Non-Steroidal Anti-Inflammatory Drug
PACTR	Pan African Clinical Trials Registry
Participant	An individual who takes part in a clinical research study
PCR	Polymerase Chain Reaction
PI	Principal Investigator
PIS	Participant Information Sheet

QMUL	Queen Mary University of London
RCT	Randomised Controlled Trial
REC	Research Ethics Committee
RSI	Reference Safety Information; the SmPC or other data provided about an IMP
RT(q)-PCR	Real-Time (Quantitative) Polymerase Chain Reaction
SAE	Serious Adverse Event
SAR	Serious Adverse Reaction
SAP	Statistical Analysis Plan
SmPC	Summary of Product Characteristics
SUSAR	Suspected Unexpected Serious Adverse Reactions
SOP	Standard Operating Procedure
SPC	Summary of Product Characteristics
TJ	Tight Junction (between intestinal epithelial cells)
TFF	TreFoil Factor
TNF	Tumour Necrosis Factor
TROPGAN	Tropical Gastroenterology and Nutrition Group
UK	United Kingdom
UN	United Nations
USD	United States Dollar (currency)
USM	Urgent Safety Measure
UNZABREC	University of Zambia Biomedical Research Ethics Committee
UTH	University Teaching Hospital, Lusaka, Zambia

WHO	World Health Organisation
ZMW	Zambian Kwacha (currency)
ZimREC	Zimbabwe Research Ethics Committee
ZIMQAP	Zimbabwe Quality Assurance Programme

9.0 Introduction

9.1 Background and literature review

Nutritional disorders are glaring examples of health inequalities between high- and low-income countries, and within low-income countries. Malnutrition underlies almost half of all child deaths globally and therefore contributes enormously to the unacceptably high under-5 mortality rates in these regions[1]. Chronic undernutrition is usually manifest as stunting (poor linear growth), affects 40% and 27% of children in Zambia and Zimbabwe, respectively (Zambia DHS 2013-14, Zimbabwe DHS 2015)[2], and is associated with increased mortality[3], reduced neurodevelopmental potential and decreased long-term economic productivity[4]. Acute malnutrition is usually manifest as wasting (loss of tissue), with or without oedema, and is the most conspicuous of all nutritional disorders. Severe acute malnutrition (SAM, for definition see section 12.1) carries the highest mortality[5], particularly if associated with medical complications. HIV has changed the epidemiology, pathogenesis and clinical presentation of SAM, and children with both conditions have a particularly high mortality[6].

Over the last two decades, three key developments in the approach to treatment have improved the outcome of SAM: standardised management protocols, ready to use therapeutic food (RUTF), and community management of acute malnutrition (CMAM).[7,8]. CMAM has four components, namely, community mobilization and community identification of cases of acute malnutrition, supplementary feeding programme (SFP) for children with moderate acute malnutrition (MAM), outpatient therapeutic programme (OTP) of SAM with no medical complications and inpatient management of SAM with medical complications. However, severely malnourished children with medical complications requiring hospitalisation[9] often fail to respond to treatment[10], and continue to experience high inpatient mortality of up to 35%[5,11]. Even after discharge, children have a poor prognosis, with 42% mortality over the subsequent year[6]. In our experience, it is a subgroup of children with SAM and acute or persistent diarrhoea who pose the most difficult management challenges, although the vast majority of children with SAM have substantial degree of enteropathy[11,12,13]. Current treatment guidelines for SAM are not well supported by an evidence base, and there is a dearth of clinical trial data; in particular, there are no specific interventions to target enteropathy in SAM[5]. In a systematic review[14], only three trials were found which inform management of SAM and persistent diarrhoea, and no trials dealing with the HIV-infected child. We therefore believe that novel therapeutic approaches are urgently needed, and that a series of small phase 2 trials could guide development of a new generation of treatments. These trials should focus on repairing damage to the small intestinal mucosa, as we now have substantial evidence that this plays a central role in the genesis of systemic inflammation, bacterial translocation and sepsis with all its adverse nutritional consequences.

Evidence of malnutrition enteropathy: Recent studies[15,16] have taught us a great deal about the infectious contribution to dysfunction of the small intestine in malnourished children. A high pathogen burden causes damage to the mucosa which exacerbates nutritional impairment and

leads to further susceptibility to infection and impaired epithelial regeneration, in a cyclical process first described in Central America in the 1970s[17,18]. This mucosal damage in SAM we refer to as ***malnutrition enteropathy***. We have been able to investigate malnutrition enteropathy in SAM. Markers of microbial translocation and systemic inflammation were dramatically increased in children with SAM compared to healthy controls[19]. Enteropathy is characterised by multiple epithelial breaches, microbial translocation from the gut lumen to the systemic circulation, and systemic inflammation (manuscripts in preparation). Epithelial breaches are present in histological sections, seen in haematoxylin/eosin-stained sections and using immunofluorescence for claudin 4 and E-cadherin[19,20]. In parallel studies in adults, we demonstrated that these lesions occur *in vivo* using confocal laser endomicroscopy[20]. We have also identified a consistent pattern of blunted epithelial repair, with reduced glucagon-like peptide 2 (GLP2) in serum, reduced trefoil factor 3 in duodenal aspirates[19], and a strong transcriptomic signature of impaired mucosal defence. We also observed two further immunological abnormalities: low-level false positive coeliac-like autoantibodies[19], and upregulation of SMAD7 similar to the pattern seen in Crohn's disease. Together, these abnormalities indicate there is substantial structural and functional damage to the small intestine, and that this primary gut pathology is associated with systemic sequelae.

WHO guidelines clearly state that antibiotic treatment should be part of the initial management of all cases of SAM, whether associated with overt features of infection ('complicated' SAM) or not ('uncomplicated' SAM)[9]. Antibiotic use was associated with reduced short term mortality and improved nutritional rehabilitation in SAM with no medical complications in Malawi[21] but post-discharge prophylaxis with cotrimoxazole did not reduce long-term mortality following complicated SAM in Kenya[22], and in Niger antibiotics conferred no benefit[10]. Even in the best outcome group in the Malawi antibiotic trial[21], mortality was not reduced below 4% over 12 weeks in children managed as outpatients in OTP. This seems to be a glass floor below which it has never been possible to reduce mortality in SAM, even in uncomplicated cases in the community[8] or in clinical trials. In SAM with medical complications who are managed as inpatients, mortality is much higher, and when combined with HIV infection can reach 35% in hospital and 62% over one year post-discharge[6].

If optimal antibiotics are insufficient to reduce mortality below 4% in uncomplicated cases treated in the community, and mortality is much higher in complicated cases, we postulate that there must be other factors which lead inexorably to adverse outcomes in a subgroup of children with complicated SAM. We and others postulate that a major contributor to the residual mortality observed after current optimal therapies have been implemented is failure of restitution in the gut. This means that in a subset of children with SAM, largely but not exclusively those with persistent diarrhoea, the intestinal mucosa is damaged and leaky, permitting efflux of plasma proteins and influx of luminal microbial products, and fails to repair following the infective insult. The evidence leading to this interpretation is:

i. In children with SAM and persistent diarrhoea, there are epithelial gaps visualised in haematoxylin/eosin-stained sections and using immunohistochemistry for claudin-4 and E-cadherin;

ii. These are morphologically similar to lesions seen in environmental enteropathy in adults, though more severe and more numerous;
iii. Plasma proteins are easily detected in western blots of duodenal aspirates, suggesting leakage from blood to gut lumen;
iv. Biomarkers of microbial products (bacterial DNA and lipopolysaccharide) are found in very high concentrations in peripheral blood, direct evidence of microbial translocation from lumen to blood;
v. Children with persistent diarrhoea and malnutrition have reduced circulating concentrations of glucagon-like peptide-2 (GLP2) and reduced trefoil factor 3 in intestinal secretions, suggesting a failure of homeostatic repair mechanisms. Circulating LPS was inversely proportional to circulating GLP2, and this remained true in multivariate analysis[19].

In a recent study of children with complicated SAM in Malawi, children who died were more likely to have diarrhoea and higher levels of faecal calprotectin (a marker of intestinal inflammation), and levels of systemic inflammation were directly related to mortality[23]. There is now clear evidence that enteropathy is associated with systemic inflammation and mortality in SAM and a new treatment paradigm is required to improve outcomes.

The need for new approaches: We postulate that the central lesion in malnutrition enteropathy is epithelial leakiness through tight junction damage and microerosions, and so we propose therapy directed at restoration of the mucosal barrier which will permit reversal of the cascade of downstream inflammatory derangements. Without novel approaches we cannot hope to achieve a radical improvement in outcome and reduce current unacceptably high mortality rates. We therefore propose four potential therapeutic approaches to achieving mucosal restoration. Colostrum, teduglutide and N-acetyl glucosamine are all intended to achieve mucosal healing. Budesonide, a corticosteroid with limited absorption, is targeted at the downstream inflammatory changes; in inflammatory conditions such as coeliac disease or Crohn's disease, steroids produce rapid clinical response. If any of these interventions impact on malnutrition enteropathy, we may open up novel therapeutic approaches which could have an impact on our world's most disadvantaged children. We may also identify where current management strategies are failing.

The novel therapeutic approaches to be tested in these trials are:

- **Colostrum** is the first liquid secreted by the lactating breast, and all breast-fed children take it for approximately the first three days of life. It is similar to breast milk, but with higher protein content. Bovine colostrum is available as a high-protein bovine colostrum powder (Colostrum UK) for use as a health-promoting nutritional supplement. The dose is 1.5g every 8 hours for 14 days, reconstituted with water and given by mouth or through a nasogastric tube. Colostrum reduces the increased epithelial permeability seen in heat shock[24]. Colostrum contains nutrients, immunoglobulins and growth factors, including epidermal growth factor (EGF) and insulin-like growth factor 1 (IGF-1).

- **N-acetyl glucosamine (GlcNAc)** is a natural sugar present on every cell surface. All breast-fed children consume GlcNAc in human milk throughout lactation[25]. It will be given orally for 14 days, gradually increased from a starting dose of 300mg to 600mg three times daily to avoid osmotic diarrhoea. Impaired glycosylation of glycosaminoglycans has been noted for many years in oedematous malnutrition, with reduced concentrations of glycosaminoglycans found in blood, urine, kidney, brain and small intestine. Specific consequences of reduced heparan sulphate expression include gut epithelial leakiness with hypoalbuminaemia[26]. GlcNAc administration has been demonstrated to restore the intestinal epithelial charged barrier in Crohn's disease[27]. GlcNAc may theoretically induce osmotic diarrhoea if not absorbed in the small intestine. Although this has not been observed clinically in animals or in older children, we will use a dose escalation schedule to minimise the chance of this happening.
- **Teduglutide** is a long-acting form of GLP2 which has proven efficacy in intestinal failure, improving absorption and reducing the need for parenteral support[28]. GLP2 is a hormone secreted by L cells in the terminal ileum, which drives epithelial repair and mediates intestinal adaptation by increased cellular proliferation and villus hypertrophy. It will be given by subcutaneous injection (0.05mg/kg/day) once daily for 14 days.
- **Budesonide** is a corticosteroid drug which reduces inflammation in the gut but is then rapidly broken down in the liver so that it does not have systemic effects. The dose is 1mg orally 8-hourly for 7 days, then 1mg 12-hourly for 4 days, then 0.5mg 12-hourly for 3 days. Budesonide is standard therapy for Crohn’s disease, and can be used for refractory coeliac disease. Since malnutrition enteropathy is characterized by intestinal inflammation, with infiltration of activated T cells[29], an anti-inflammatory approach is rational. A prior trial of mesalazine in Kenya confirmed an immuno-modulatory approach is safe in the setting of SAM[30], but targeting the small, rather than large, intestine with a more potent agent is likely to be more effective.

9.2 Assessment and management of risk

Risks associated with this trial may arise from two sources, the IMPs, or the trial procedures required to evaluate efficacy or safety. Professor Playford, Professor Murch and Dr Hill have direct experience of these IMPs, and they will contribute that experience to safety monitoring. The IMPs are:

- **Colostrum** Bovine colostrum is potentially allergenic in theory, though there is no evidence that cow’s milk products are harmful in this patient group and the standard therapeutic feeds for these children (F75 and F100 recommended by WHO) contain milk proteins. Professor Playford was senior author of the reported use of bovine colostrum to reduce intestinal permeability in adults[24], and will advise and assist with monitoring of adverse events.
- **N-acetyl glucosamine (GlcNAc)** theoretically might induce osmotic diarrhoea, though this has not been reported in animal studies or in clinical practice. Professor Murch has experience of use of GlcNAc in the context of a clinical trial in children with inflammatory bowel disease[27] and is responsible for the SOP for administration. Dose escalation will be used to minimise the risk of diarrhoea (see section 15.13).
- **Teduglutide** is usually given for months or years. In a recently published description of tolerability of 12 weeks of teduglutide in children in the UK and the USA[31], vomiting was attributed to teduglutide in 10% of recipients and pyrexia in 14%. No children had treatment withdrawal because of adverse events. Three adverse events were considered of special interest (intestinal obstruction, fluid overload and biliary derangements) but none were

observed. Dr Susan Hill was co-author of this report and is responsible for the SOP for administration; she will assist with review of suspected adverse events.

- **Budesonide** may cause immunosuppression and other corticosteroid effects (oedema, hypertension, glucose intolerance, osteoporosis), but usually only after longer term administration. It is the corticosteroid of choice for intestinal disorders because it causes fewer adverse events than prednisolone (see BNF for children, section 1.5) because of low systemic absorption. Professor Murch and Dr Hill have extensive experience of using this drug in children with Crohn's disease and will be available for consultation about any AEs where advice is needed. The dose will be tapered to mitigate any possible effects of adrenal suppression.
- All children, including those in the **standard care** arm, will receive standard care based on WHO guidelines for management of complicated SAM[9]. For standard of care procedures, please follow this link: https://www.who.int/elena/titles/full_recommendations/sam_management/en/

Study procedures also contribute to risk:

- **Blood collection** may cause pain and bruising at the site of venepuncture. The trial medical and nursing staff are highly experienced, which will minimise these risks. Ametop gel, EMLA cream or any other substitute will be offered to reduce discomfort.
- **HIV testing** may cause distress if a positive result is disclosed. Testing will be carried out according to Zambian and Zimbabwean guidelines. Fully trained counsellors will be responsible for pre- and post-test counselling as per usual practice in Zambia and Zimbabwe, and will be available to support nursing staff.
- **Endoscopy** carries well-defined risks. **It will be performed in the endoscopy unit in Lusaka only, not in Harare**. American Society for Gastrointestinal Endoscopy (ASGE) guidelines provide a clear framework concerning the workup of children with malnutrition/failure to thrive who fail to respond to nutritional rehabilitation and in whom no apparent cause of malnutrition can be identified after basic lab workup[32], as practiced in our research to date. This may reveal a treatable, secondary cause of malnutrition including coeliac disease, occult or opportunistic infections associated with growth failure[33]. Endoscopy of eligible children will be performed in the UTH endoscopy unit which has an excellent safety record. Endoscopies will be performed by Dr Amadi, Professor Kelly and Dr Chandwe, and sedation/general anaesthesia will be provided by trained anaesthestists using ketamine-based anaesthesia.

Counselling for the recommended treatment for coeliac disease will be provided to the families of affected children in local language and identified infections will be treated according to the standard of care. Follow up will be continued to facilitate treatment and ensure best possible outcome depending on pathology identified.

During the endoscopy, we will employ a new technique to allow confocal laser endomicroscopy in children, which has until now only been possible in adults. This is the Cellvizio system made by Mauna Kea, and we have available a system designed specifically for children using Pentax gastroscope (external diameter 8mm). To get these images we will inject fluorescein intravenously (1-2ml of a 2-4% solution, just as in retinal angiography in ophthalmology departments all over the world). The procedure, which is shorter and simpler than that used in adults, will take 2 minutes. We have used confocal endomicroscopy in 235 adult volunteers and patients in the UTH endoscopy unit with no adverse events at all. We have recently extended this experience to 45 children, again with

no adverse events.

The endoscopy, with a maximum of 6 small intestinal biopsies for analysis as specified in section 13.8.3, will therefore provide a thorough assessment of efficacy and safety as we will be able to detect any deterioration.

9.3 Rationale for study design

There is emerging awareness that multi-arm Phase 2 clinical trials offer a more efficient approach to identification of new therapies than parallel conventional trial designs, in which one agent is evaluated against a control. Our recent work on the pathophysiology of malnutrition enteropathy suggests that mucosal healing may be central to reducing mortality rates, but we do not yet know the efficacy of several plausible therapeutic approaches. In order to evaluate these four new potential therapies efficiently and quickly, they will be compared side-by-side using biomarkers of pathophysiology as endpoints. This design has the following advantages:

i. The four novel therapies will each be evaluated against standard care;
ii. This evaluation will run side-by-side, so that all children will be randomised to any of the five groups at any point in time to avoid biases due to seasonal variation;
iii. The trial endpoints will evaluate a range of biomarkers that capture different domains of malnutrition enteropathy, to allow a comprehensive non-invasive assessment of mucosal healing
iv. A subgroup of children will have endoscopy (only in Lusaka), where the endoscopy unit has a track record of safe endoscopy in children;
v. Endoscopic biopsy will provide evaluation of healing and ensure that the selected therapy does not induce unwanted immune or other effects;
vi. The overall trial design provides an efficient way of identifying one or more candidates for a Phase 3 trial.

10.0 Trial flowchart

Children hospitalised with SAM will be enrolled from three hospitals once they have completed the stabilization phase of nutritional rehabilitation and are clinically stable. Children will be randomised in an allocation ratio of (1:1:1:1:1) to either Colostrum, GlcNAc, Teduglutide, Budesonide, or standard care only, in addition to the standard care already provided.

Randomisation codes will be prepared in advance by the trial statistician, and treatment allocation will be revealed after enrolment by opening sealed envelopes held by the trial coordinators in each of the three study sites. There will be no attempt at blinding as the interventions are readily distinguishable (e.g. teduglutide is given as subcutaneous injection, so to mask all interventions would require administration of several placebo formulations); however, the primary endpoints are biomarkers so laboratory staff and data analysts will be blinded to randomised group. In future trials of any promising interventions, the timing of introduction of novel therapies would be an explicit research question, as they may also be of value earlier in treatment. However, for this phase II trial, we will only enrol children once they are clinically stable. The sequence of interventions is described in Figure 1

Figure 1 Flow of patients through the trial

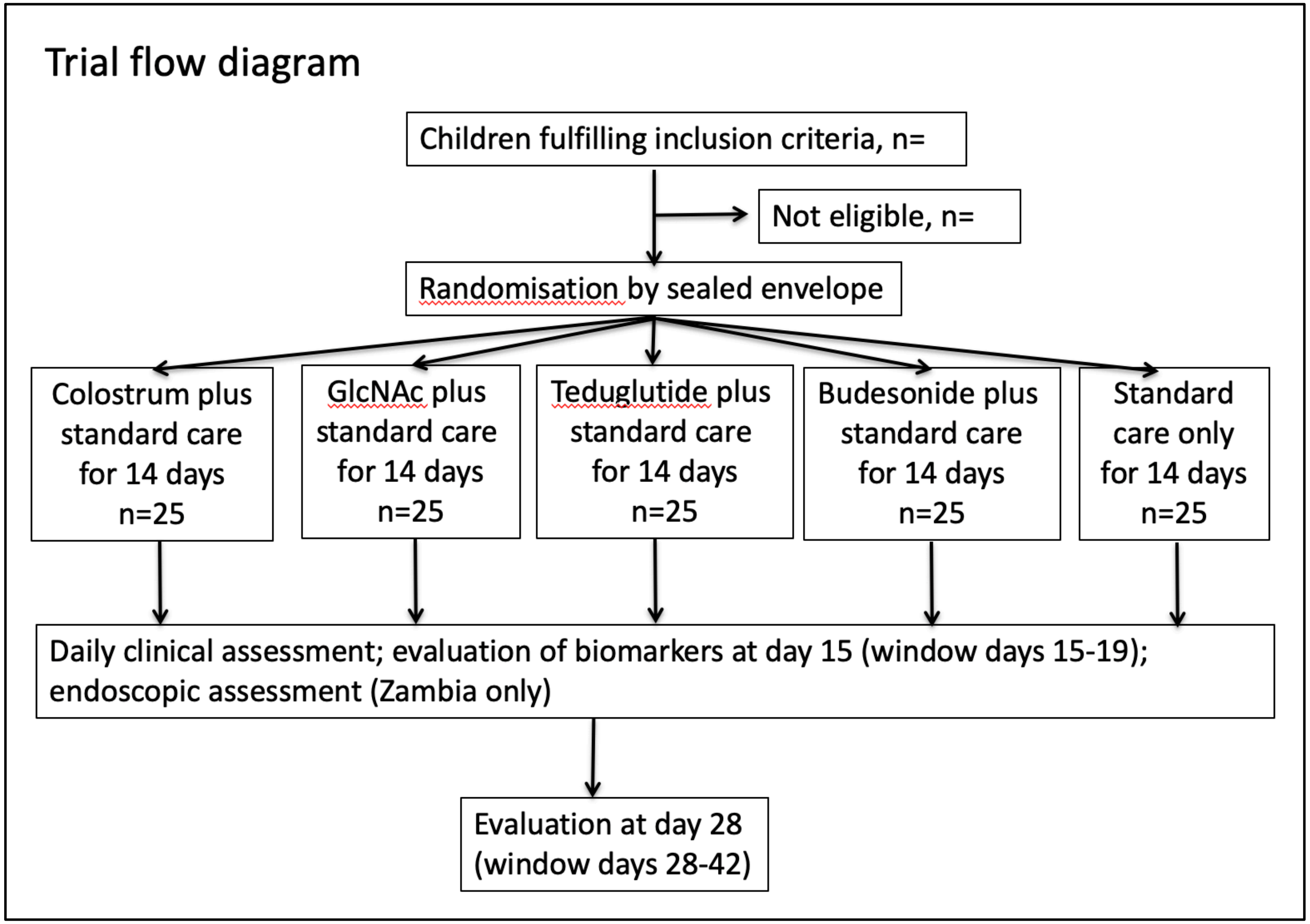

***Window period at end of study: 15-19 Days**

11.0 Trial Objectives and Design

11.1 Primary Objective/s

The hypothesis to be tested is that at least one of the four interventions will improve malnutrition enteropathy over 14 days by restoring mucosal integrity, reducing intestinal inflammation, lowering microbial translocation and reducing systemic inflammation.

All children will receive standard care based on WHO guidelines for in-patient management of complicated SAM[9]. This includes the ten steps approach to nutritional rehabilitation, standard use of antibiotics, standard micronutrient supplements, and standardized feeds (F75, F100 and peanut-based ready-to-use therapeutic food: RUTF). All four interventions, given together with standard care, will be compared against standard care only.

The primary objective is to determine if any of the four novel therapies (budesonide, colostrum, N-acetyl glucosamine, or teduglutide) delivered alongside standard care can improve malnutrition enteropathy in African children with severe acute malnutrition. This will be evaluated using non-invasive biomarkers of intestinal damage: mucosal permeability, mucosal inflammation and regeneration, microbial translocation and systemic inflammation.

11.2 Secondary Objective/s

To assess clinical and nutritional recovery;

To evaluate the impact of the interventions on biomarkers of pathophysiology;

To explore the difference in response between HIV infected and uninfected children.

In a subset, mucosal healing will be evaluated at endoscopy.

11.3 Endpoints

11.3.1 Primary Endpoints

The primary endpoints for this trial will be measured on day 15 (allowable window 15 to 19 days) after initiating treatment by analysis of faecal biomarkers. Details of endpoint measurements are described in section 13.8.3, and analysis in section 19. Gut inflammation will be measured as a composite score of faecal myeloperoxidase, neopterin and alpha-1 antitrypsin. This composite score is derived from the work of Kosek et al[34], incorporating the

weightings used in that score, and will be calculated as:

Enteropathy biomarker score = 2x(AAT in mg/g) + 0.2x(MPO in µg/ml) + 1x(neopterin in µmol/l)

11.3.2 Secondary Endpoints

Secondary endpoints will be measured by daily clinical review (for clinical endpoints) during the intervention period and by collection of biological specimens on day 15 (allowable window 15 to 19 days) after initiating treatment.. All biomarkers will be measured as described in section 13.8.3, and presented as median and interquartile range (IQR).

i. Plasma intestinal fatty acid binding protein (FABP) concentration (ng/ml);
ii. Plasma lipopolysaccharide (LPS; EU/ml);
iii. Plasma LPS binding protein (LBP; ng/ml);
iv. Plasma soluble CD14 and CD163 (µg/ml);
v. Plasma C-reactive protein (mg/l);
vi. Plasma albumin (g/l);
vii. Mortality by day 15 and day 28;
viii. Change in anthropometric measurements (weight, mid upper arm circumference, weight-for-height) between baseline and day 15, and baseline and day 28;
ix. Resolution of oedema between baseline and day 15;
x. Adverse events between baseline and day 15;
xi. Serious adverse events between baseline and day 15;
xii. Days with diarrhoea between baseline and day 15;
xiii. Days with fever (two or more recordings of axillary temperature of 37.5^{o}C by or higher in any 24 h period) between baseline and day 15;
xiv. Hormones: GLP-2, IGF-1 and IGFBP3 (all in ng/ml);
xv. Morphometric measures on biopsy specimens collected in Zambia only: villus height (µm), crypt depth (µm), villus width (µm), epithelial surface

perimeter (µm), and inflammatory scores;

xvi. Lactulose and rhamnose recovery (Lusaka only).

11.4 Exploratory or Tertiary endpoints/outcomes to explore mechanisms of any observed effect on secondary endpoints

i. Glycosylation status measured in saliva and biopsies by Western blotting/immunohistochemistry
ii. Urine and plasma metabolomics, both untargeted and targeted to citrulline and Kyn/Trp ratio;
iii. Transcriptomic analysis of intestinal biopsies;
iv. Mucosal leakiness measured by observation of plumes and luminal fluorescein during confocal laser endomicroscopy at day 15 (range 15-19 days).
v. Changes in other inflammatory or endothelial activation biomarkers, measured from the plasma

11.5 Objectives and End Points Summary

Primary Objective	*Endpoint*	*Outcome Measures*
Determine if interventions can improve malnutrition enteropathy	Recovery of mucosal integrity	Enteropathy biomarker score (faecal myeloperoxidase, alpha1-antitrypsin, neopterin)
Secondary Objectives	*Secondary Endpoints*	*Outcome Measures*
Biomarker concentrations		Plasma fatty acid binding protein (FABP)
		Plasma lipopolysaccharide
		Plasma LBP
		Plasma soluble CD14, CD163
		Plasma C-reactive Protein
		Plasma albumin
		Hormones: GLP-2, IGF-1 and IGFBP3
Clinical course		Mortality by day 15 and day 28
		Adverse events, including clinical events and laboratory toxicity evaluated by full blood count and biochemistry
		Serious adverse events

		Days with diarrhoea
		Days with fever
		Change in weight, weight-for-height and MUAC between baseline and day 15
		Days with oedema (children with oedematous SAM only)
Intestinal mucosal assessment (Lusaka only)		Villus height, crypt depth, villus width, epithelial surface area, inflammation score
		Lactulose and rhamnose recovery
Tertiary Objectives	*Tertiary Endpoints*	*Outcome Measures*
Exploratory work on glycosylation		Glycosylation in saliva and biopsies
Metabolomics		Untargeted metabolomic profile by mass spectroscopy, and specific measurement of citrulline, tryptophan and kynurenine
Transcriptomic analysis		Untargeted analysis of mRNA sequencing data from intestinal biopsies
Confocal laser endomicroscopy		Mucosal leakiness measured by observation of plumes and luminal fluorescein during confocal laser endomicroscopy
Biomarker concentrations		Inflammatory markers: CCL3, CCL4,

		CCL11, GCSF, GM-CSF, IFN-gamma, IL-1b, IL-1ra, IL2, IL-6, IL-8, IL-10, IL-33, TNF-a
		Endothelial markers: Angiopoetin-1, D-dimer, EGF, ICAM1, IGFBP-3, PIGF, CD62L, CD62P, TPO, VCAM1, VEGF

11.6 Trial design

This is a multi-arm Phase II trial designed to evaluate if any of four potential therapies for malnutrition enteropathy could be taken forward into Phase III trials which would include clinical recovery and mortality as primary outcomes.

11.7 Study setting

The greatest burden of malnutrition is in Africa and south Asia. This trial will directly address malnutrition in one of these settings by conducting the trial in two African cities with a high burden of malnutrition, both of which have considerable experience in treating malnutrition-related disorders in children. Both have well-equipped wards, experienced staff and well-equipped laboratories.

12.0 Eligibility Criteria

12.1 Inclusion Criteria

1. Age 6 – 59 months, of either sex;
2. Inpatient in the paediatric wards of one of the research sites;
3. Hospitalised with Severe Acute Malnutrition (SAM, defined using WHO definition: weight-for-length z score of less than -3, or mid upper arm circumference of less than 11.5cm, and/or bilateral pedal oedema);
4. Clinically stable*;
5. With written, informed consent from the primary caregiver(s); the child cannot be enrolled if the primary caregiver(s) cannot give consent.

*Judged by the medical team on a case by case basis, but in general a child without shock, hypothermia, hypoglycaemia or reduced conscious level.

12.2 Exclusion Criteria

1. Clinically unstable*;
2. Less than 5kg body weight;
3. Neurological disability which would explain or partly explain poor feeding;
4. Oro-facial abnormalities which would explain or partly explain poor feeding;
5. Caregiver unwilling to consent to child HIV testing;
6. Haemoglobin concentration < 6 g/dl at the time of enrolment;
7. Caregiver unwilling to remain in hospital for the duration of the study treatment;
8. Any underlying condition, other than HIV, which in the opinion of the investigator would put the subject at undue risk of failing study completion or would interfere with analysis of study results;
9. Contraindication to any of the trial treatments (e.g. allergy to cow's milk protein).

*As assessed by the medical team on a case-by-case basis, but in general a clinically unstable state would include shock, hypothermia, hypoglycaemia or reduced conscious level.

13.0 Trial procedures

13.1 Recruitment

Children will be recruited on the paediatric wards of the three hospitals: Children's Hospital in UTH, Harare Central Hospital, and Parirenyatwa Hospital. We have considerable experience of enrolment into studies at these sites[8,11,19] and will use similar approaches for this trial. All children are screened on admission to hospital for SAM, and study staff will review all new admissions each day to identify potentially eligible children as explained in section 13.2. Caregivers of all children will be sensitized to the study using a sensitization leaflet, so that all caregivers on the ward are aware of the study.

13.2 Participant identification

Initial screening will be carried out by study staff on the wards of the hospitals in Zambia and Zimbabwe. This will be carried out during the stabilisation phase. This will include:

- An eligibility check using information already available, with written permission from the parents/caregivers, with confirmation of accuracy of anthropometric measurements;
- Provision of participant information sheet (PIS) and full discussion of what the study involves;
- Leading up to written informed consent.

13.3 Informed Consent Procedures

Written, informed consent will be obtained from the primary caregiver(s) of all participating children. A copy of the written information sheet and consent record will be provided and retained by each family. Parents or guardians who wish to understand what happens to the samples their child will donate may be invited to a demonstration of the laboratory facilities. This has worked well in the past to allay concerns about the intended purpose of obtaining specimens such as blood or intestinal biopsies[35].

13.3.1 Responsibility for obtaining consent

The Principal Investigator (PI) retains overall responsibility for the informed consent of participants at their site and will ensure that any person delegated responsibility to participate in the informed consent process is duly authorised, trained and competent to participate according to the ethically approved protocol, principles of Good Clinical Practice (GCP) and Declaration of Helsinki. Delegation of consent will be detailed in the Site Delegation Log.

Consent will be taken by a medical practitioner or nurse named in the study team and delegation log. When consent is taken by a person other than a medically qualified health worker, a doctor will confirm and document that the patient meets all eligibility criteria.

13.3.2 Consent Considerations

The right of a participant to refuse participation without giving reasons will be respected, as stated in the informed consent form.

The participant will remain free to withdraw at any time from the trial without giving reasons and without prejudicing his/her further treatment

and will be provided with a contact point where he/she may obtain further information about the trial; this will be the PI in each hospital. Where a participant is required to re-consent, for example if during the trial new Research Safety Information becomes available, or following an amendment that affects the patient, or new information needs to be provided to a participant, the PI accepts the responsibility of ensuring this is done in a timely manner.

13.3.3 Population

Children with SAM in the paediatric wards of the study hospitals.

13.3.4 Vulnerable participant considerations

Participants in this trial will be children under 5 years of age. These are vulnerable participants from the consent perspective, so the greatest care will be taken to ensure that parents and guardians will understand the risks and benefits of participation fully. The PI takes responsibility for ensuring that all vulnerable participants are protected and participate in an environment free from coercion or undue influence.

13.3.5 Written/reading/translation considerations

The information sheet will be translated formally into local languages, and all versions will be made available throughout the trial.

13.3.6 Participants lacking capacity

All participants will be children, and the consent procedures are described above. It is assumed that parents/guardians will have full capacity to give consent for the children under their care. In Zimbabwe, mothers aged between 15 and 18 years who are considered “emancipated minors” will be allowed to give consent. Consent for such research in Zambia and Zimbabwe is usually considered a family affair, so family discussions are expected and encouraged.

13.3.7 Minors

All participants will be children under 5 years of age, so consent will be obtained from primary caregivers and assent will not be sought.

13.3.8 Consenting process

Trial nursing and medical staff will identify eligible children whose primary caregivers will then be approached to begin the process of discussing the trial and the written, translated, information sheet in the most appropriate language. The consent process will include honest discussions of risks and benefits of the interventions, the concept of randomisation, and the purpose and intended use of samples collected, which is an important issue in this trial as the primary endpoints are all laboratory biomarkers. The consent process will continue throughout the trial, but all parents/guardians will sign to record that they have given informed consent to their child's participation, before any study procedures commence.

We have pioneered the use of participant visits to laboratories to facilitate valid consent[35]. This opportunity will be afforded to these parents and guardians. At least 24 hours will be available for consideration of consent; in practice, 2-3 days is the norm in Zambia and Zimbabwe because mothers/carers usually need to discuss enrolment with other family members.

The written information sheet, the translations, and the consent record will all be approved by the ethics committees in each country (UNZABREC and MRCZ).

13.3.9 Additional consent provisions for collection and use of participant data and biological samples in ancillary studies

In Zimbabwe, a separate consent sheet will be provided for recording consent for long-term storage and shipment of samples. In Zambia, the Health Research Act 2013 provides for the setting up of biobanks, but these provisions are not yet in force and for the moment biobanking is not possible. The National Health Research Authority will make these provisions in due course; if this becomes possible during the conduct of this trial we will arrange for the regulations to be followed.

13.4 Screening procedures

Screening in Lusaka will be carried out routinely as all malnourished children are together in one ward (ward A07). In both hospitals in Harare, children are not on the same ward, so trial nurses and doctors will engage with all paediatric teams to find children who are eligible. New admissions will be screened each morning to identify potentially eligible children, whose caregivers will be approached for consent as outlined in section 13.3.

13.5 Patient allocation

13.5.1 Randomisation Method

A randomisation list will be generated by the trial statistician in a ratio of 1:1:1:1:1 using simple block randomisation with a random variable block length.

13.5.2 Randomisation Procedure

Due to constraints on the available infrastructure, workload of staff and the emergency nature of the illness, it is not feasible to use an online randomisation scheme. Sealed envelopes will be used to randomise the participants. To ensure allocation concealment, the envelopes will be opaque to ensure the allocated treatment cannot be determined prior to formally randomising the patient.

Randomisation will be stratified by site, and separate envelopes will be provided to UTH, Parirenyatwa and Harare Central Hospitals.

Once informed consent is obtained from a patient, baseline measurements will be taken (see section 13.8.2) and then randomisation will occur to one of the treatments: Colostrum, N-acetyl glucosamine (GlcNAc), Teduglutide, Budesonide or Control (Standard Care). Final consent and randomisation will not take place until the child with consenting parents/guardians has reached the point where he/she is considered stable (defined as above).

Staff on the wards will record the number of patients randomised into the trial through patient ID stratified by site. Sites will have emergency back-up randomisation envelopes to be used in the event that the operating envelopes are not accessible, in case of damage or missing

envelopes. If emergency back-up envelopes are required, the staff will continue by selecting the next sequential patient number within the site from the list.

13.5.3 Cohort allocation/sequential allocation

The allocation sequence will be generated by the trial statistician. Enrolment of participants and assignment to interventions will be carried out by medical and nursing staff on the wards.

13.6 Blinding

The trial will not be blinded as the different treatments are difficult to mask. However, the primary endpoints are laboratory-based and laboratory staff and data analysts will be blinded to treatment allocation during assay performance and analysis. Samples will be run and analysed using trial ID only, with all data cleaning and re-assays carried out blinded. The trial statistician will unblind lab data once databases are finalised.

13.7 Unblinding

None of the primary endpoints have clinical relevance so unblinding will not be needed. All AEs will be evaluated clinically and appropriate clinical investigations carried out as indicated.

13.8 Trial schedule

13.8.1 Schedule of Treatment for each visit

All contacts between the study team and children/parents/caregivers will occur in a hospital ward. Using the convention that day 1 is the day on which treatment commences:

Day -5 to 0: Screening of children (see section 13.2) and initial approach to parents/caregivers to provide basic information and invite further discussions; provision and explanation of full participant information sheet. Contacts at this stage will be recorded on a Screening Questionnaire which will also record

written consent to the child's assessment for eligibility.

Day -2 to 1: Written, informed consent obtained and randomisation carried out.

Day -2 to 1: Baseline assessment after formal consent obtained. Questionnaire administered, physical examination and anthropometry completed, blood taken for initial samples and HIV test (if not already conducted as part of clinical care); urine, saliva (subset only) and stool sample collection.

Day 1: Allocated treatment commenced; Investigational product administration recorded.

Days 1-4: Daily clinical review and physical examination.

Day 5: Daily clinical review and physical examination; safety blood tests (haematology, biochemistry) recorded on CRF form 8.

Days 6-15: Daily clinical review and physical examination.

Day 15-19: Efficacy blood tests. A stool sample, urine sample and saliva sample (subgroup) will be collected. In Lusaka, endoscopy will be arranged on one of days 15-19.

End of study (day 28-42): Questionnaire and physical examination and anthropometry. If not possible, phone consultation only.

13.8.2 Schedule of Assessment (in Diagrammatic Format)

Assessment	Screening From admission	Enrolment and randomis-ation (days -2 to 1)	Baseline assess-ment (days -2 to 1)	Days 1-4	Day 5	Days 6-14	Days 15-19	End of Study (days 28-42)
Eligibility assessment and initial approach about possible enrolment; provision of PIS	x							
Signature on consent record		x						
Questionnaire, Physical Examination & Anthropometry			x				x	x
IMP				x	x	x		
Clinical review and physical				x	x	x	x	

examination								
Blood (HIV test)			x					
Blood (research)			x				x	
Blood (safety)			x		x		x	
Saliva (Lusaka only)			x				x	
Urine			x				x	
Stool			x				x	
Endoscopy and biopsy, with lactulose rhamnose recovery (Lusaka only)							x	

"Safety" blood samples include FBC and biochemistry (U&Es, LFTs, PO_4).

13.8.3 Trial assessments

All trial personnel from Lusaka and Harare will be trained at study initiation in study procedures, questionnaire administration, and sample collection procedures.

Questionnaires (CRF forms 3,12 and 14): a brief questionnaire will be administered by a trial nurse to obtain relevant demographic and clinical information about the child.

Physical examination including anthropometry: Physical examination will include routine clinical observations. Weight, length/height, mid-upper arm circumference will be measured using standardised techniques following training according to the anthropometry SOP. Anthropometry will

be repeated on day 15.

Blood samples: Blood will be collected from children using a syringe and needle or butterfly, using universal precautions. Blood (maximum 4 ml on any one occasion; total amount not to exceed 2 mL/kg over 2 weeks) will be collected into endotoxin-free EDTA/heparin tubes. Blood biomarkers, and volumes of plasma required for testing are shown in the table.

<table>
<tr><td>Blood biomarker</td><td>Full name</td><td colspan="2">Function/rationale</td><td>Volume required</td></tr>
<tr><td>LPS</td><td>Lipopolysaccharide</td><td colspan="2">Component of bacterial wall of Gram negative bacteria; presence in blood implies translocation of microbes (or their components) from the gut</td><td>50 ⍰l</td></tr>
<tr><td>LBP</td><td>Lipopolysaccharide binding protein</td><td colspan="2">Released on binding of LPS to monocyte-lineage cells through TLR4</td><td>110⍰l</td></tr>
<tr><td>FABP</td><td>Intestinal fatty acid binding protein</td><td colspan="2">Required for absorption of fatty acids. Intestinal type (type II) is only present in enterocytes and release into bloodstream implies gut damage</td><td>25⍰l</td></tr>
<tr><td>sCD14</td><td>Soluble CD14</td><td colspan="2">Part of signalling system for detection of LPS by TLR4 on macrophages and related cells</td><td>3⍰l</td></tr>
<tr><td>CD163</td><td>CD163</td><td colspan="2">Released by activation of Kupffer cells and related macrophage lineage cells</td><td>5⍰l</td></tr>
<tr><td>CRP</td><td>C-reactive protein</td><td colspan="2">Inflammatory marker</td><td>20⍰l</td></tr>
<tr><td>GLP-2</td><td>Glucagon-like peptide 2</td><td colspan="2">Hormone released by L cells of ileum which drives mucosal regeneration in response to malabsorption</td><td>50⍰l</td></tr>
<tr><td>IGF-1</td><td>Insulin-like growth factor 1</td><td colspan="2">Signalling molecule/hormone which mediates effect of growth hormone on growth plate of child bones</td><td>20⍰l</td></tr>
<tr><td>IGFBP3</td><td>IGF-binding protein 3</td><td colspan="2">Responsible for carrying IGF-1 in the circulation</td><td>10⍰l</td></tr>
<tr><td>Metabolomics</td><td>Metabolomics</td><td colspan="2">Full spectrum of small molecules analysed by NMR</td><td>50⍰l</td></tr>
<tr><td colspan="2">Multiplex array</td><td>Multiple</td><td>Multiplex analysis of multiple markers of inflammation and endothelial activation, which will help to explain the</td><td>50⍰l</td></tr>
</table>

		results seen in the secondary endpoints.<br>Inflammatory markers: CCL3, CCL4, CCL11, GCSF, GM-CSF, IFN-gamma, IL-1b, IL-1ra, IL2, IL-6, IL-8, IL-10, IL-33, TNF-a<br>Endothelial markers: Angiopoetin-1, D-dimer, EGF, ICAM1, IGFBP-3, PIGF, CD62L, CD62P, TPO, VCAM1, VEGF	
Total volume required if all assays run			373 l

Stool samples will be collected for faecal myeloperoxidase, neopterin, and 1-antitrypsin assays. Saliva will be collected in Lusaka only for glycosylation analysis (Secretor (FUT2) status at baseline, and thereafter to monitor the response to GlcNAc by western blotting for glycosylated proteins. Urine will be collected for 3 hours after endoscopy, following instillation of lactulose 5g and rhamnose 1g in 10ml during endoscopy.

Endoscopic biopsies to be collected in Lusaka will be used as specified below:

Biopsy	Processing	Test	Testing Group	Hypotheses Tested
1-2	FFPE	H&E and Immunofluor-escence staining	TROPGAN lab	Mucosal morphometry, epithelial breaks identified using claudin 4, E-cadherin, occludin, and expression of TFF3 and EGFR.
3-4	Snap frozen in liquid N_2	RNA sequencing or other questions of interest	BGI or TROPGAN lab	Agnostic analysis of transcriptome, targeted analysis of defence pathways
5	Carnoy's solution or frozen	Analysis of mucins	TROPGAN lab	Thinning of mucus layer and alteration of composition can be modified by interventions

6	Fresh,<br>20 minute incubation	Lactase and disaccharide test	TROPGAN lab	Lactase deficiency for clinical care

13.8.4 Follow up Procedures

Children will be seen daily for research purposes, and more often if clinical problems require. Primary endpoint measurements will be complete by day 19 (target date 15 days post-treatment initiation; window of 15-19 days). Children will be invited to attend for a final, end of study, visit on or about day 28 (window 28-42 days).

13.8.5 Qualitative assessments – Nested studies

Caregivers will be asked 3-4 simple questions on their views about the acceptability of the therapies during the day 28 review. The short questionnaire will be administered by study nurses and reviewed to identify any concerns on the part of caregivers about the interventions their children have received.

13.8.6 Radiology Assessments

n/a

13.9 Withdrawal criteria

In the event of an SAR, the child will be withdrawn. In all cases a SAR will be discussed with the TMG within 24h and, exceptionally, a decision might be made to continue treatment in view of these circumstances of the case. A child may also be withdrawn from the trial if, in the opinion of the TMG, an event has occurred which necessitates withdrawal in the child's best interests. Caregivers are free to withdraw their children from the trial at any time.

13.10 Early withdrawal

If early withdrawal occurs at the instigation of one of the trial committees, data and specimens will still be retained, and will contribute towards primary endpoint analysis.

As stated in the informed consent forms, caregivers can withdraw from all or part of the study at any time. We will ask caregivers who wish to withdraw to sign an exit form which states whether they wish to withdraw from part of the study (for example, specimen collection) or from the whole study.

13.11 End of trial (EOT)

The CI is delegated the responsibility of submitting the EOT notification to all relevant regulatory bodies and Sponsor. The EOT notification must be received by these bodies and Sponsor within 90 days of the end of the trial. If the study is ended prematurely, the Chief Investigator will notify these bodies and Sponsor, including the reasons for the premature termination (within 15 days).

14.0 Laboratories and samples

14.1 Central Laboratories

Zambia: TROPGAN is a research group situated in the Department of Internal Medicine at University Teaching Hospital, Lusaka. There are five laboratories with dedicated space for PCR preparation and cycling (3-room set up), immunoblotting and electrophoresis, flow cytometry (BD FACSverse), trace element analysis (ICP spectrometry, Perkin Elmer), ELISA, and immunohistochemistry. Samples are received at room temperature or in liquid nitrogen and archived in -20 or -80°C freezers using standard operating procedures. The laboratory operates according to the principles of Good Laboratory Practice. Immunohistochemistry, Sample processing and storage, and ELISAs will be conducted at TROPGAN.

Zimbabwe: Zvitambo has a purpose-built 5-room laboratory, with facilities for PCR, flow cytometry (BD FACSVerse), ELISA assays, CD4 count testing and cell culture. The lab has a customized laboratory information system (LIS) for tracking sample receipt and archiving. The laboratory operates according to the principles of Good Laboratory Practice, with appropriate quality control (ZIMQAP) procedures and use of standard operating procedures, and is overseen by the laboratory manager. Sample processing and storage, and ELISAs will be conducted at Zvitambo. Luminex multiplex biomarker processing will also be carried out in Zvitambo due to the availability of the highly specialised machine, running a specific biomarker panel.

In order to ensure quality and comparability across study sites, assays for primary and some secondary endpoints may be conducted in only one of the two laboratories. For other endpoints, standards will be aliquoted and run across study sites simultaneously with pooled QC samples to ensure comparability. Details will be specified in the Laboratory SOP.

14.2 Local Laboratories

Samples for safety analysis (haematology and biochemistry) will be processed in the laboratories of the University Teaching Hospital, Lusaka, or in Parirenyatwa hospital, or in approved local laboratories.

14.3 Sample Collection/Labelling/Logging

Blood samples will be collected into the following tubes provided by the trial, as follows:

4 ml: for haematology and plasma/serum separation. The cellular fraction will be stored for flow cytometry.

Samples will be stored in cooler boxes and transported to the laboratory within 2h, then centrifuged at 4000 rpm at 4°C prior to aliquoting and storage.

Saliva will be collected into 2ml Cryovials for FUT2 and glycosylation analysis.

Urine and stool will be collected into plain 30ml universal containers.

Samples will be labelled with ID number and date only (thus pseudononymised). Samples (blood, saliva, urine, stool) will be logged and signed for in the ward, upon collection and when dispatched to the laboratories, then again when received, analysed, stored or destroyed. Endoscopic biopsies will be treated in the same manner.

For safety reasons, the maximum blood draw will be 2ml/kg body weight over a 2 week period.

14.4 Sample Receipt/Chain of Custody/Accountability

Upon receipt in the laboratory, samples will be divided into research samples and clinical safety samples. All research samples will be identified by barcode labels only. A sample transmittal form is used to ensure chain of custody, with signatures from the research nurse, porter/driver and laboratory technician to ensure the sample has arrived in the laboratory.

14.5 Sample Analysis Procedures

14.5.1 The arrangements for sample analysis

Samples for research assays will be analysed immediately or stored at appropriate temperature (usually -80°C) until batches can be run. Excess sample will be stored at -80°C, and spare aliquots retained wherever possible as back-up in case of analytical failures, or storage failures during transport to another laboratory. Samples for safety analysis (haematology and biochemistry) will be processed immediately; excess sample will be retained as back-up but destroyed once the study is complete.

14.5.2 Sample Storage Procedures

Barcoded samples will be stored at -80°C (+/- 10°C), as FFPE tissue for immunohistochemistry, or as extracted DNA at room temperature. Freezer temperatures will be checked daily.

In the event of withdrawal of consent, the withdrawing participant will be asked whether their samples may continue to be used; if so, the samples will be retained, but if not the sample will be destroyed.

14.6 Sample and Data Recording/Reporting

Data will be recorded in written reports and on laboratory computers as pseudononymised (ID code only) with no identifiable information. These will be transferred electronically to data capture computers used for data entry. Data will be collated between Harare and Lusaka. No identifiers will be captured electronically. There are no commercial partners with whom data might be shared.

14.7 End of study

Samples will be stored while the ethics approval for the study is current, at which point the pseudononymous samples will be destroyed or archived. Samples to be destroyed will be disposed of as clinical waste in accordance with GLP.

14.8 Transportation of samples internationally

For samples from Lusaka or Harare requiring transport overseas for analysis, a material transfer agreement (MTA) will be prepared for transferring samples between Zambia/Zimbabwe and the overseas laboratory, according to the requirements of the National Health Research Ethics Committee of the Zambian Ministry of Health and the Medical Research Council of Zimbabwe. Import and export of samples will comply with national and international regulations. After any overseas assays and experiments are complete, the samples will either be returned to TROPGAN / University of Zambia School of Medicine facilities in Zambia or to the Zvitambo Institute for Maternal and Child Health Research in Harare, or destroyed. Consent will be obtained for long term storage in Zimbabwe, but such storage in Zambia will only be allowed if TROPGAN is successful in obtained a biobank licence before ethics committee approval expires. Samples from HIV seronegative participants will be packaged, labelled and transported as 'Exempt human specimens', in accordance with UN / WHO and IATA regulations. Samples (apart from blood) from HIV- seropositive participants (processed biopsies; processed blood samples such as protein extracts; faeces; processed duodenal aspirates) are not considered infectious and will be packaged, labelled and transported as 'Exempt human specimens', in accordance with UN / WHO and IATA regulations.

FFPE tissue may be sent to London for immunohistochemistry for those molecules which cannot be imaged in Lusaka or Harare. Biopsies for RNA sequencing will be collected for single cell transcriptomic analysis or bulk transcriptomics depending on the results of preliminary work ongoing at the Massachusetts Institute of Technology (recipient details will be confirmed).

<u>Sender (and recipient) details: Zambia</u>
Ms Kanekwa Zyambo,
Laboratory manager, TROPGAN
University of Zambia School of Medicine,
University Teaching Hospital,
Nationalist Road,
Lusaka, Zambia

<u>Sender (and recipient) details: Zimbabwe</u>
Ms Kuda Mutasa
Laboratory manager

Zvitambo Institute For Maternal And Child Health Research
16 Lauchlan Ave, Meyrick Park,
Harare, Zimbabwe

Recipient details: London
Dr Debbie Hampson,
Institute Lab Manager, Blizard Institute
4, Newark Street, London E1 2AT, UK

Recipient details for metabolomics analysis: Imperial College, London

Dr Jonathan Swann
Level 5, Sherfield Building
Imperial College
South Kensington
London SW7 2AZ, UK

15.0 Trial Interventions

15.1 Name and description of investigational medicinal product(s)

1 Teduglutide: This is marketed as Revestive in the UK and Gattex in the USA. This is provided as liquid formulation for subcutaneous injection. Teduglutide is currently supplied as a powder that is dissolved in a diluent (supplied by Takeda) and injected s-c. Each single-use glass vial contains either 5 mg or 1.25mg of teduglutide as a white, lyophilized powder for reconstitution with 0.5 mL Sterile Water for Injection provided in a prefilled syringe; the 1.25mg vial is to facilitate dosing in children under 15kg body weight.

2 Budesonide: Budesonide liquid, as respules.

15.2 Name and description of the nutritional products

Both colostrum and N-acetyl glucosamine are dietary constituents which almost all children ingest; they are generally regarded as safe and freely available as food supplements in health shops.

1 Colostrum: Bovine colostrum bulk powder, to be re-packaged into dose capsules by Colostrum UK.

2 N-acetyl glucosamine: N-acetyl glucosamine bulk powder, to be re-packaged into dose capsules by Colostrum UK.

15.3 Legal status of the products

1 Colostrum: nutraceutical, generally regarded as safe, not licensed as a medicine.

2 N-acetyl glucosamine: nutraceutical, generally regarded as safe, not licensed as a medicine.

3 Teduglutide: licensed in Europe and USA for children with intestinal failure.

4 Budesonide: provided as liquid from respules; the liquid is not licensed for use as an oral agent but is in common use for this indication.

15.3 Summary of Product Characteristics (SmPC) or IB

See Appendices. Note that there is no reference safety information for Colostrum or N-acetyl glucosamine. Any serious adverse reactions to these products will be deemed unexpected.

15.4 Storage and supply

All the IMPs and nutritional products will be procured from suppliers by Barts health pharmacy, London E1 2AT. Drugs will be stored in the Pharmacy of the hospital in which it is intended to be dispensed. For details of dispensing arrangements see Pharmacy SOP.

15.5 Supplier

All the IMPs and nutritional products will be procured from suppliers by Pharmacy, Barts Health NHS Trust, London E1. Colostrum and N-acetyl glucosamine may have been encapsulated by Colostrum UK, but additional batches may be supplied to pharmacy as powders. The individual suppliers are:

1 Colostrum: Colostrum UK

2 N-acetyl glucosamine: Blackburn Distributors

3 Teduglutide: Takeda Pharmaceuticals

4 Budesonide: Alliance Healthcare

15.6 Manufacturer

1 Colostrum: Colostrum UK

2 N-acetyl glucosamine: Blackburn Distributors

3 Teduglutide: Takeda

4 Budesonide: Alliance Healthcare

15.7 How the products should be stored

Products will be stored in the Pharmacy (or other designated space) of all three hospitals: UTH, PH and HCH. Teduglutide and budesonide should be stored between 4 and 8°C. Colostrum and N-acetyl glucosamine should be stored between 4 and 25 ^{o}C Storage conditions on the ward will be specified in the Pharmacy SOP.

15.8 Details of accountability

Products will be shipped from Barts Health and stored in the Pharmacy in each hospital. A register will be kept of the products as received. Products will be dispensed for each patient by a registered pharmacist and an accountability log kept of all product dispensed, the amount and date.

15.9 Destruction/return and Recall

Products not used by the end of the trial, or expired before that date, will be destroyed in the hospital incinerator and recorded as such in the IMP register by the pharmacist. At the end of the trial a certificate of destruction will be issued by each pharmacist.

15.10 Prescription of IMP / nutritional product

Products will be prescribed on bespoke charts specific to the TAME trial. Children allocated to Standard Care will have no additional prescriptions.

15.11 Preparation and labelling of IMP and nutritional products

Each container will be labelled with a label designed and affixed by the local pharmacies at the three study sites, as specified in the Pharmacy SOP.

15.12 Preparation and Administration of IMP and nutritional products

Instructions will be provided for each of the medications as follows:

1 Colostrum:

Powder to be dissolved in water and then to be given orally or via NG tube every 8 hours.

2 N-acetyl glucosamine

Powder to be dissolved in water and then to be given orally or via NG tube every 8 hours.

3 Teduglutide

Give by subcutaneous injection once daily. Rotate injection sites.

4 Budesonide

Liquid to be given orally or via NG tube every 8 or 12 hours.

15.13 Dosage schedules

All investigational products are to be given for 14 days

**Group**	**A**	**B**	**C**	**D**	**E**
Product	Colostrum	N-acetyl glucosamine	Teduglutide	Budesonide	Standard care
Presentation	Powder in capsules	Powder in capsules	Ampoules	Liquid	-
Number of patients	25	25	25	25	25
Dose:<br>days 1-7<br>days 8-11	 1.5g tds<br>1.5g tds	 300mg tds<br>600mg tds	 0.05 mg/kg daily<br>0.05 mg/kg daily	 1mg tds<br>1mg bd	 -<br>-

days 12-14	1.5g tds	600mg tds	0.05 mg/kg daily	0.5mg bd	
No of capsules or vials	7,560	2835	315	2880	-
Weight of IMP (kg)	2.835	0.850	0.394	1.440	-

15.14 Dispensing of product

Products will be administered by the trial nurses on the wards. Dispensing of all product will be recorded in an accountability log.

15.15 Dosage modifications

The dose of N-acetyl glucosamine may be reduced if the child experiences a worsening of diarrhoea, as a precaution in case this might be exacerbated by an osmotic effect in the gut lumen. The dose of colostrum may need to be reduced if the child experiences a worsening of diarrhoea, as colostrum contains small amounts of lactose and some children may have lactase deficiency. However, to mitigate this we will procure reduced-lactose colostrum. Any IMP dosage modifications or interruptions will be recorded on CRF form 5 together with the reason for them. A dose of teduglutide may be omitted if vomiting or troublesome posseting develops.

15.16 Known drug reactions and interaction with other therapies

None known.

15.17 Prior and Concomitant medication

There are no restrictions on concomitant medication. All children will receive broad-spectrum antibiotics, mebendazole and vitamin A according to WHO guidelines[9]. Children with diarrhoea will receive oral rehydration therapy and zinc according to WHO guidelines[9].

15.18 Trial restrictions

None

15.19 Assessment of compliance

By nursing report, as recorded on the IMP daily record (CRF form 5).

15.20 Name and description of each Non-Investigational Medicinal Product (NIMP)

Standard Care will be provided for all children in the trial, including those allocated to one of the four IMPs. This includes all the ten steps of the WHO protocol[9], and thus all children will receive broad-spectrum antibiotics, selected according to current microbial resistance patterns.

Fluorescein will be given to children during endoscopy, to permit confocal laser endomicroscopy. Fluorescein is used as an intravenous fluorophore in retinal angiography, with abundant evidence of safety, and we have experience of this in children with SAM, with no adverse events to date. We have used fluorescein for this indication in adults and children with no adverse effects.

15.21 Arrangements for post-trial access to IMP and care

It is not anticipated that children will receive any IMP after day 14. The philosophy behind treatment of severe acute malnutrition is that it is a reversible disorder and that the child will recover spontaneously if supported during the acute illness. The TAME trial aims to accelerate this recovery, and no specific treatment should be required once recovery is under way. The study team will ensure that any non-IMP treatments, which are part of standard care for SAM, will be provided for each child at and beyond discharge.

16 Equipment and Devices (used optionally during endoscopy; Zambia site only)

IN THIS SECTION OUTLINE WHAT EQUIPMENT AND/OR DEVICES ARE TO BE USED IN THE TRIAL.	
Reason for use of equipment in the trial	To provide additional information about the leakiness of the small intestinal mucosa, to establish efficacy and confirm safety
Device Name	Cellvizio laser endomicroscopy system, with Alveoflex probes
Description of the device	Confocal laser endomicroscopy system for high resolution imaging of the epithelial lining of the small intestine.
Manufacturer Name	Mauna Kea Technologies
CE Mark Number	CE0459
Device being used within its licensed purpose?	Yes
Length of time the device has been in use	6 months
Is the Device on loan or gift to the site/sponsor?	No

If the device is on loan or gifted is the device registered?	Not applicable
Initial equipment/Device Training and calibration	Training carried out in May 2016 by the manufacturer. This instrument re-calibrates before every use.
Device storage requirements and location	Clean, dry conditions in the UTH endoscopy unit
Device and equipment custodian	The CI is the custodian.
Device Maintenance requirement	No requirement, though annual service is suggested.
NB: Does the company providing or loaning the device want the study data? No	

17 Pharmacovigilance

17.1 General Definitions

Term	Definition
Adverse Event (AE)	Any untoward medical occurrence in a participant to whom a medicinal product has been administered, including occurrences which are not necessarily caused by or related to that product.
Adverse Reaction (AR)	An untoward and unintended response in a participant to an investigational medicinal product which is related to any dose administered to that participant.<br><br>The phrase "response to an investigational medicinal product" means that a causal relationship between a trial medication and an AE is at least a reasonable possibility, i.e. the relationship cannot be ruled out.<br><br>All cases judged by either the reporting medically qualified professional or the Sponsor as having a reasonable suspected causal relationship to the trial medication qualify as adverse reactions.
Serious Adverse Event (SAE)	A serious adverse event is any untoward medical occurrence that:<br><br>• Results in death.<br>• Is life-threatening.<br>• Requires inpatient hospitalisation or prolongation of existing hospitalisation<br>• Results in persistent or significant disability/incapacity.

	• Consists of a congenital anomaly or birth defect.<br><br>Other 'important medical events' may also be considered serious if they jeopardise the participant or require an intervention to prevent one of the above consequences.<br><br>NOTE: The term "life-threatening" in the definition of "serious" refers to an event in which the participant was at risk of death at the time of the event; it does not refer to an event which hypothetically might have caused death if it were more severe.
Serious Adverse Reaction (SAR)	An adverse event that is both serious and, in the opinion of the reporting Investigator, believed with reasonable probability to be due to one of the trial treatments, based on the information provided.
Suspected Unexpected Serious Adverse Reaction (SUSAR)	A serious adverse reaction, the nature and severity of which is not consistent with the information about the medicinal product in question set out in the Reference Safety Information (RSI):<br><br>• In the case of a product with a marketing authorisation, in the summary of product characteristics (SmPC) for that product.<br><br>• In the case of any other investigational medicinal product, in the investigator's brochure (IB) relating to the trial in question.

17.2 Site Investigators' Assessment

All participants will have daily clinical review by a trial physician who will specifically evaluate the child for any possible adverse events,

particularly those that may be related to the IMP or nutritional product. All adverse events will be reported in real time to the trial team and evaluated as outlined below.

The Principal Investigator who is responsible for the care of the participant, or in his/her absence an authorised medic within the research team, is responsible for assessment of any event for:

- Seriousness

  Assessing whether an AE is serious according to the definitions given in section 17.1.

- Causality

  Assessing the causality of all AEs in relation to the IMP or nutritional product according to the definition given. If an SAE is assessed as being definitely or probably related to the product, then it is defined as a SAR.

- Expectedness

  Assessing the expectedness of all AEs according to the definition given and with reference to this protocol and the RSI (see section 17.3). If the SAR is unexpected, then it is a SUSAR. All AEs will be classified by the study team in terms of their likely relationship to the IMPs and nutritional products. The classification is set out below:

Score	Causal Relationship	Definition
1	Definitely	The AE follows in a reasonable temporal sequence from product administration and, in the opinion of the investigator is definitely causally linked to the treatment.
2	Probably	The AE follows a reasonable temporal sequence from product administration, and cannot be reasonably explained by the subject's clinical state. The degree of certainty with respect to causality is less than that described above.
3	Possibly	The AE follows a reasonable temporal sequence from product administration or could have been produced by the subject's clinical state or by other modes of therapy administered to the subject.

4	Remote	The temporal relationship is such that the product would not have had any reasonable association with the observed event.
5	Definitely Not	The AE was definitely produced by the subject's clinical state or by other therapies administered to the subject.

- Severity

  Assessing the severity of the event according to the following terms and assessments. The intensity of an event should not be confused with the term “serious” which is a regulatory definition based on patient/event outcome criteria.

  - **Mild**: Some discomfort noted but without disruption of daily life
  - **Moderate**: Discomfort enough to affect/reduce normal activity
  - **Severe**: Complete inability to perform daily activities and lead a normal life

17.3 Reference Safety information

Reference Safety Information (RSI) is the information used for assessing whether an adverse reaction is expected. The Summary of Product Characteristics (SmPC) for Teduglutide and Budesonide, will be used to determine whether adverse reactions are expected; see Appendix. There is no RSI for Colostrum and N-acetyl-glucosamine. Any serious adverse reactions to colostrum or N-acetyl glucosamine will be considered unexpected.

17.4 Notification and reporting Adverse Events or Reactions

Notification and reporting will follow the flow chart below

Adverse event reporting for use of compliance team

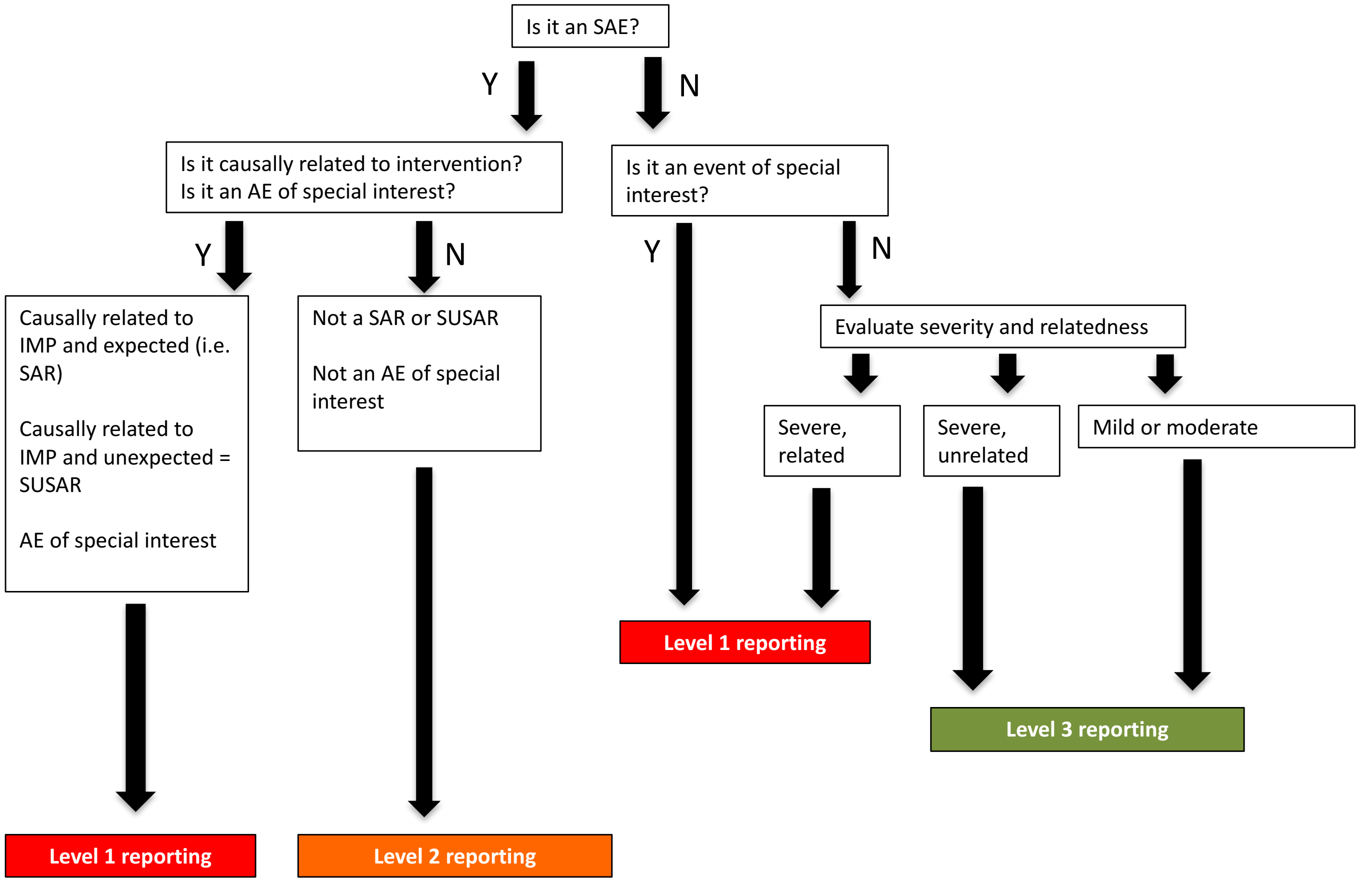

All adverse events will be recorded immediately on Form 15 and reviewed within 24 hours (or next working day) by the study team. The AE will also be documented in the participants' medical notes. For all AEs, a follow-up form will be completed to ensure resolution of all events.

17.5 Notification of AEs of special interest

An AE of special interest is an adverse event that should be reported expeditiously. These should be reported to the CI for discussion with the TSC and DMEC. For N-acetyl glucosamine and colostrum, osmotic diarrhoea will constitute an AE of special interest. For teduglutide, the following will constitute AEs of special interest:

- fluid overload;
- intestinal obstruction;

as these have been reported in adults during long-term treatment (though not in recent reports in children[29]).

17.6 Adverse events that do not require reporting

None. All AEs should be reported.

17.7 Notification and Reporting of Serious Adverse Events & SUSARs

Three reporting procedures will be adopted, reflecting the level of seriousness, severity and relatedness of the AE to the trial intervention. These are:

Level 1: Reports sent within 24 hours to Sponsor, and either UNZABREC (Zambia) or MRCZ (Zimbabwe). Then individually listed in monthly reports to Sponsor, DMEC, UNZABREC, ZAMRA, MRCZ/MCAZ and TMG. In exceptional cases the CI may decide to advise DMEC within 24 hours.

Level 2: Reports sent within 24 hours to Sponsor, and in Zimbabwe to MRCZ within 3 days. Then individually listed in monthly reports to Sponsor, DMEC, UNZABREC,ZAMRA, MRCZ/MCAZ and TMG.

Level 3: Summary tabulation in monthly reports to Sponsor, DMEC, UNZABREC, ZAMRA, MRCZ/MCAZ and TMG.

All Serious Adverse Events (SAEs) will be recorded in the participants' notes and reported on a trial Event Form 15 and reviewed by the PI or deputy within 24 hours. All SAEs will be reviewed for causality, expectedness and severity as defined in section 17.2. Death as a result of disease progression is expected in 10-20% of children with SAM in hospitals in Zambia and Zimbabwe, so up to 45 deaths are expected in this population of sick, hospitalised children due to the effects of malnutrition. Nominated co-investigators (as listed) will be authorised to sign the SAE forms in

the absence of the PI at the participating sites.

17.8 Sponsor Medical Assessment

The Sponsor has delegated the responsibility for oversight of IMP safety profile and medical assessment of SAEs and SUSARs to the CI. The CI or delegate must review all SAEs within 24 hours of receipt. This review should encompass seriousness, severity, relatedness and expectedness. Day 0 for all SUSARs is when the SAE/SUSAR is received by the CI and /or coordinating team and /or sponsor whichever is first. **It is expected that the CI will achieve oversight of IMP safety profile through trial committees as per section 28.0.**

It is noted that the CI can upgrade an event to 'related' or 'unexpected' but cannot downgrade the PI assessment of an event to 'unrelated' or 'expected'. If there is disagreement between CI and PI assessment, it is the CI's responsibility to liaise with the Site PI before CI's final decisions. The CI and PI assessment can differ.

17.9 Urgent Safety Measures

The CI may take urgent safety measures to ensure the safety and protection of the clinical trial subjects from any immediate hazard to their health and safety, in accordance with Regulation 30. The measures should be taken immediately. In this instance, the approval of the Competent Authority prior to implementing these safety measures is not required. However, it is the responsibility of the CI to attempt, where possible, to discuss the proposed changed with the Sponsor (via telephone) prior to implementing the change if possible.

The CI has an obligation to inform both the Sponsor, the local Research Ethics Committee and competent authorities **in writing within 3 days**, in the form of a substantial amendment. The sponsor (JRMO) must be sent a copy of the correspondence with regards to this matter as soon as it is sent.

17.10 Procedures for reporting blinded SUSARs

n/a

17.11 Pregnancy

n/a

18.0 Annual reporting

Development Annual Safety Update (DSUR)

The DSUR will be written by the CI (using Sponsor template) and submitted to the Sponsor for review. The DSUR is due for submission within 60 days of the end of the reporting period. The reporting period is annually from the date on the “notice of acceptance letter”. As delegated Sponsor Medical Assessor the CI will carry out a risk benefit analysis of the IMPs encompassing all events having arisen on the trial. All RECs will be sent a copy of the DSUR.

Annual Progress Report (APR)

The APR will be written by the CI and submitted to the sponsor for review prior to submission to the REC. The APR is due within 30 days of the anniversary date of the “notice of acceptance letter” letter from the Sponsor.

19.0 Statistical and Data Analysis

19.1 Sample size calculation

Kosek et al[34] examined established markers of intestinal inflammation which can be used to obtain population-based measures of intestinal injury and altered intestinal function. They developed an environmental enteropathy (EE) activity score which is a composite score of three stool markers for mucosal inflammation. The composite score is derived from measurements of faecal myeloperoxidase (MPO), neopterin (NEO) and alpha1-antitrypsin (AAT). These are stable compounds, biologically inert and highly resistant to proteolysis, therefore suitable as outcomes collected in the field.

The weighted EE score is given in Equation 1:

Equation 1
EE score = 2 x (AAT category) + 2 x (MPO category) + 1 (1 x NEO category)

We will use the principal of a weighted enteropathy score as our primary outcome. We will not trichotomise the variables as Kosek et al[34] did, but keep the score as a continuous composite variable[37]. This is because we are interested in gauging the potential magnitude of the effect these treatments have, and applying a cut of values to a continuous composite value could mask or discard information[37]. The modified Enteropathy score is given in Equation 2, with modification of weighting of units to bring measurements into a comparable arithmetical range:

Equation 2
EE biomarker score = 2x(AAT in mg/g) + 0.2x(MPO in µg/ml) + 1x(neopterin in µmol/l).

We will have a control arm, so can understand the effect of the treatment against a reference standard.

There are no available data from our represented population to draw upon for an understanding of the baseline variance of the EE biomarker score. The primary outcome response from each patient is the EE biomarker score at 14 days (window 14-18 days) after treatment initiation, adjusted for baseline values. We have assumed that the EE biomarker score is normally distributed with a common standard deviation. To determine the sample size, we drew upon two methods to establish an effect size. We assumed the difference would be larger than the likely inherent imprecision in the measurements of each outcome, and we have defined the magnitude of the effect on a standardised scale. The trial will detect a medium/large effect of Cohen's d effect size of 0.3, with 80% power and 90% confidence, and a conservative correlation between baseline and follow-up estimate of 0.5, we will need a sample size of 23 per group across 5 groups to analyse with the ANCOVA method. We

expect there to be approximately 5% loss to follow up due to deaths another reasons, such as drug intolerance or withdrawal. Adjusting our sample size of 115 for 5% loss to follow-up and rounding up to a multiple of 5, we aim to randomise 125 patients in total (25 in each group). We will evaluate our withdrawal rate again and adjust the sample size accordingly towards the end of the trial.

19.2 Planned recruitment rate

At least one child per week in each site (i.e. total recruitment rate of 3 per week).

19.3 Statistical analysis plan (SAP)

For continuous variables, we will remain unblinded and examine the distribution of the data for symmetry, and report either the mean and SD, or the median and interquartile range (IQR) values. A separate SAP will be designed by the three trial statisticians and the TMG. Salient features are described below.

19.4 Summary of baseline data and flow of patients

We will report baseline measurements by treatment group with summary statistics only, and in line with CONSORT guidance, no statistically significant testing of baseline characteristics will occur[38]**.** To generate the trial CONSORT flow diagram, the initial cell will include all children assessed for eligibility, whether or not eligible and whether or not their caregivers gave consent.

19.5 Primary outcome analysis

Population set

The primary analysis will use the per-protocol population set. Participants who experience a protocol violation (to be defined in the SAP) will be excluded from the population set. Patients who die during treatment will be excluded from the primary analysis set. We will report the number of deaths and mortality rate of patients per arm, and test if there is a difference in mortality rate to the control for each treatment group.

Statistical Analysis primary endpoint:

The four treatment groups will be compared to the control. We will use a mixed effect ANCOVA model on the environmental enteropathy for activity score against the control adjusting for the core covariates stated below. Treatment effects will be deemed statistically significant if the p-value is less than or equal to 0.1 when compared to the control arm, for all 4 treatments.

We will not undertake any adjustments of the false positive (type I) error rate, as the aim of this trial is to inform the treatment development process for this population, which would lead to a Phase III trial if a degree of efficacy is observed. The general consensus is that adjustment for the type I error rate is not required in exploratory multi-arm multi-stage trials in Phase II within the treatment development framework[39].

If patients are discharged from hospital sooner than day 14, we will measure the primary endpoints as close as the discharge date as possible, and carry this observation forward as the primary outcome.

19.6 Secondary outcome analysis

We will provide summary tables on the crude primary and secondary outcomes by treatment group, reporting p-values and estimated 95% confidence intervals of treatment on the variables. Secondary outcomes which are mechanistic will be evaluated on a mixed effect linear model, and adjusted for the core covariates given above. We will report mortality rate in all treatment arms, and withdrawals due to clinical instability and the number of patients who discharge early in each arm.

19.7 Subgroup analyses

None.

19.8 Adjusted analysis

For both primary and secondary outcomes, we will adjust for a core set of baseline variables. These are: sex (male/female); oedema (yes/no); HIV status (yes/no); diarrhoea (yes/no); baseline WLZ scores (continuous); baseline biomarker/histology scores (continuous) and site. Study site is the stratification variable. Through due diligence, the TAME trial team aim to have complete capture of all data from all patients. Further details of adjusted analysis will be provided in the SAP.

19.9 Interim analysis and criteria for the premature termination of the trial

There will be no interim analysis.

19.10 Subject population

The analysis will include the protocol-compliant population as this is a Phase II trial.

19.11 Procedure(s) to account for missing or spurious data

No attempt will be made to account for missing data. Spurious data (e.g. implausible values) will be corrected while the participant is on the ward, or dropped.

19.12 Other statistical considerations

None

19.13 Economic evaluation

n/a

20.0 Data Handling & Record Keeping

20.1 Confidentiality

Information and data related to participants will be kept confidential and managed in accordance with the requirements of UNZABREC and MRCZ. Paper records with personally identifiable information (questionnaire front sheet; study register/logbook) will be stored in a secure, locked cupboard at each site and will be kept fully confidential. HIV and endoscopy test results are confidential. With the participant's consent, HIV and endoscopy results will be documented in his / her confidential medical notes for use by other health care professionals. Results cannot be shared with anyone outside the study team without the caregiver's explicit consent. Data will be entered onto the study databases without personally identifiable information. Database data will be linked to the paper records through patient study identification number only (pseudo-anonymisation). Study samples (blood; stool; biopsies), including ones for transport to / from Zambia and Zimbabwe, will be labelled with patient study identification number, date of collection, and specimen type only.

20.2 Data Custodian Details

The Chief Investigator will be the Custodian of the research data:

Professor Paul Kelly

The identifiable information to be collected will include:

- Name
- Date of birth
- Address and location details of family residence
- Phone numbers of mother/parents/guardian and secondary contact as appropriate.

These data will be recorded on a front sheet attached to the trial file for each participant. The data will also be recorded in a trial register.

Clinical teams will have access to this information while the child is in the trial. At the end of the trial period (day 41 at the latest), these details

will be detached from the file which will then be sent for data entry, and the identifiable information will be stored in a locked cupboard at each site. At the end of the study follow up (day 28) the personal identifiable data (which will never have been recorded electronically) will be retained in a locked cupboard so that the consent form for each sample or dataset can be verified. Data will be retained for up to 20 years.

20.3 Pseudononymisation

The study ID code given upon enrolment will be used as the identifier on all paper records, computer records and samples. Each child will be given a screening ID number which will be distinct from the trial ID number. The only exclusion criterion that requires laboratory data is haemoglobin (<6 g/dl) but this is routinely measured as part of clinical care for children admitted with SAM and will not be undertaken as a study screening test. The study ID code will therefore not contain any patient identifiable components such as initials.

20.4 Transferring/Transporting Data

Data may be shared between investigators electronically, but always in an unlinked form. At no stage will personal identifiable information be entered onto a database of any sort.

20.5 Data collection tools and source document identification

All study files will be de-identified after follow-up and data entry are complete. All identifiers (consent form, locator data) will be stored separately in a locked filing cabinet and not entered onto any electronic database. Source data will be kept securely for 20 years.

20.6 Source Data

CRFs, in hard copy, will constitute the source data. Laboratory records will be only identified by ID number; where necessary for clinical care, data will be copied into clinical records.

20.7 Case Report Form

The CRF will include baseline clinical and demographic data, the eligibility (inclusion and exclusion) checklist, record of dispensed IMPs, AE, SAE and SUSAR record forms, daily follow-up data, biosample checklist, and exit form. These forms are summarised in the Table below.

Form	Form Title
1	Screening consent form
2	Screening form
3	Baseline questionnaire
4	Baseline investigations checklist
5	Daily IMP record
6	Randomisation
7	Daily clinical review
8	Daily investigations summary
9	Day 5 investigations summary
10	Day 14 investigations summary
11	Endoscopy record
12	Discharge, including questions on acceptability of interventions
13	Day 28 evaluation
14	Premature exit and withdrawal
15	Adverse events
16	Death

20.8 CRFs as Source Documents

CRFs will be considered as the primary source records and all data will be entered onto these directly by the medical and nursing team. Laboratory records will be stored separately, and ELISA printouts and direct records preserved as source documents. These will be signed as authentic records by all laboratory

staff according to GLP precepts. Similar procedures will cover endoscopy records, which will be completed after every procedure. In the endoscopy unit, separate records using only the ID number will be kept as source documents.

20.9 Data handling and record keeping

All trial participants are children in hospital wards. All data will be collected by trial doctors and nurses, and by laboratory staff; all records will be signed and dated on completion to enable checks to be carried out by the trial coordinator and trial monitors. A central register will be kept which will link CRFs to consent records. Data will be entered by trained staff onto a Redcap-based database (or database with similar functionality), twice, followed by resolution of all discrepant entries against CRFs. Redcap tracks all entries, thus enabling audit.

20.10 Access to Data, Source Data and Documents

Direct access will be granted to authorised representatives from the Sponsor, host institution and the regulatory authorities to permit trial-related monitoring, audits and inspections.

Only the clinically qualified members of the research team will have access to the CRF while the identifiable information is still attached. Laboratory staff will have access to records generated in the laboratory only. Once entered and pseudononymised, all named investigators across study sites will have access. Members of the laboratory team will have access only at the end of the trial once the code is broken, unless required for academic (e.g. PhD) work, in which case the CI or PIs may release partial datasets without treatment allocation data.

21.0 Archiving

Records will be kept for a further 20 years after the conclusion of the trial. Destruction of essential documents will require authorisation from the Sponsor.

22.0 Monitoring, Audit and Inspection

22.1 Monitoring

Monitoring procedures are detailed in the Trial Monitoring Plan.

22.2 Auditing

Sponsor retains the right to Audit any trial, trial site or central facility. In addition, any part of the trial may be inspected by the regulatory bodies and funders where applicable.

22.3 Notification of Serious Breaches to GCP and/or the protocol

A serious breach is defined as a breach which is likely to affect the safety or physical or mental integrity of a participant, or the scientific value of the trial. The Site Principal investigator is responsible for reporting any serious breaches to the Sponsor **within 24 hours.** The Chief Investigator is responsible for reporting any serious breaches to the Sponsor **within 24 hours**. The Sponsor will work with the CI to investigate any potential breach within 7 working days of becoming aware of a serious breach.

22.4 Compliance

The CI will ensure that the trial is conducted in compliance with GCP guidelines, the World Medical Association Declaration of Helsinki (1996), the Sponsor's SOPs, legislation in Zambia and Zimbabwe, and other regulatory requirements as amended.

22.5 Non-Compliance

Protocol deviations will not be permitted. Accidental deviations will be investigated and corrective action taken. Non-compliances may be captured from a variety of different sources including monitoring visits, CRFs, communications and updates. The sponsor will maintain a log of the non-compliances to ascertain if there are any trends developing which need to be escalated. The CI and the coordinating team should assess the non-compliances and action a timeframe in which they need to be dealt with. This assessment should include the need to escalate to the sponsor. Any event with the potential to affect participant safety or data integrity must be reported to the sponsor within 24 hours of the coordinating team becoming aware. Where applicable corrective and preventative actions should be assigned. Each action will be given a different timeframe dependent on the severity. If the actions are not dealt with accordingly, the Sponsor will agree an appropriate action, which could include an on-site audit.

22.6 Regulatory Compliance

The trial will not commence at each study site until a Clinical Trial Authorisation (CTA) is obtained from the relevant authority: the Zambia Medicines Regulatory Authority (ZAMRA) or the Medicines Control Authority of Zimbabwe (MCAZ). This study does not involve ionising radiation.

23.0 Financial and other competing interests for the chief investigator, PIs at each site and committee members for the overall trial management

None of the Investigators or collaborators named in this protocol have any financial interest in any of the IMPs named in this protocol.

24.0 Ethical and Regulatory Considerations

Before the start of the trial, approval will be sought from the Research Ethics Committees (RECs) and national regulatory authorities for the trial protocol, informed consent forms and other relevant documents.

The decision whether any amendment constitutes a minor or substantial amendment lies with the Sponsor. Substantial amendments that require review by the Sponsor and RECs and national regulatory authorities (where relevant) will not be implemented until these authorities grant favourable opinion for the study.

All correspondence with the Sponsor, RECs and national regulatory authorities will be retained in the Trial Master File at the lead site and Investigator Site File at each site.

The Chief Investigator will notify the RECs, the Zambia Medicines Regulatory Authority (ZAMRA), the Medicines Control Authority of Zimbabwe (MCAZ), and Sponsor of the end of the study.

25.0 Peer review

This trial was peer-reviewed as part of the grant awarding process by the Medical Research Council, UK and will be peer reviewed as part of the ethics review process in each country.

26.0 Public and Participant Involvement

Public participation in trial design has not been considered possible. We have actively pursued the idea of participant visits to laboratories in the past and will make this available to caregivers of participants in the TAME trial.

27.0 Indemnity

27.1 Amendments

If the sponsor wishes to make a substantial amendment to the CTA or the documents that supported the original application for the CTA, the sponsor must submit a valid notice of amendment to the national regulatory authorities and to the RECs for consideration. It is the Sponsor's responsibility to decide whether an amendment is substantial or non-substantial. Amendments also need to be notified to participating sites to assess whether the amendment affects the approvals relevant to that site. All amendments will be version-tracked.

27.2 Access to the final trial dataset

All of the members of the TMG will have access to the final dataset.

28.0 Trial Committees

Three committees will be constituted.

The Trial Management Group will comprise the CI and PIs and those key clinically-qualified investigators involved in study design. The TMG and statistician will meet electronically, or wherever possible face-to-face, at least monthly or more often in the event of issues relating to safety. The TMG will have the power to terminate the trial, but these decisions will be heavily influenced by the TSC and the DMEC. Such decisions will be taken by consensus wherever possible, but the final decision will rest with the TMG. Full minutes will be taken and will be made available to the Sponsor after every meeting.

The Trial Steering Committee will comprise two independent paediatricians and an independent statistician. The charter of the TSC will be to advise on the progress of the trial, which it will do every 6 months in response to a progress report from the TMG.

The DMEC will comprise one or two independent paediatricians and one or two independent statisticians. The charter of the DMEC will be to advise on the progress of the trial, which it will do every 6 months in response to a progress report from the TMG. The DMEC will also review any SAEs or SUSARs, with a particular emphasis on searching for trends suggesting unexpected adverse events.

All committee members will complete declarations about potential conflicts of interest, GCP training and their CVs will be retained for monitoring.

29.0 Publication and Dissemination Policy

29.1 Publication

Publications will be prepared at the end of the trial. Appropriate study reports will be made available on the clinical trial website in which the trial is registered. All publications will acknowledge the Sponsor and all participating institutions. The results will be published on a publically accessible database within 2 years of the end of the trial.

29.2 Dissemination policy

Dissemination meetings will be held in all study centres (Lusaka and Harare) at the end of the trial and all possible attempts will be made to invite caregivers of participants in the trial. Publications will be authored by the members of the TMG and others by invitation according to the criteria for authorship of the ICMJE. The funding agency will be acknowledged in the publications arising, but will have no role in the dissemination/publication processes. Publication will be Open Access. Professional medical writers will not be employed. Databases (fully anonymised) will be shared only after the TMG have decided by consensus that their manuscripts are complete.

30.0 References

1 Black RE, Victora CG, Walker SP, Bhutta ZA, Christian P, de Onis M, Ezzati M, Grantham-McGregor S, Katz J, Martorell R, Uauy R. Maternal and child undernutrition and overweight in low-income and middle-income countries. Lancet. 2013; 382: 427-51.
2 Zambia Demographic and Health Survey 2013-14; Zimbabwe Demographic and Health Survey 2015. Rockville, Maryland, USA: Central Statistical Office, Ministry of Health, and ICF International.
3 Olofin I, McDonald CM, Ezzati M, Flaxman S, Black RE, Fawzi WW, Caulfield LE, Danaei G. Associations of suboptimal growth with all-cause and cause-specific mortality in children under five years: a pooled analysis of ten prospective studies. PLoS One 2013 8: e64636.
4 Victora CG, Adair L, Fall C, Hallal PC, Martorell R, Richter L, Sachdev HS. Maternal and child undernutrition: consequences for adult health and human capital. Lancet 2008; 371: 340–357.
5 Lenters LM, Wazny K, Webb P, Ahmed T, Bhutta ZA. Treatment of severe and moderate acute malnutrition in low- and middle-income settings: a systematic review, meta-analysis and Delphi process. BMC Public Health. 2013; 13 Suppl 3: S23.
6 Kerac M, Bunn J, Chagaluka G, Bahwere P, Tomkins A, Collins S, Seal A. Follow-up of post-discharge growth and mortality after treatment for severe acute malnutrition (FuSAM study): a prospective cohort study. PLoS One 2014; 9: e96030.
7 Collins S, Dent N, Binns P, Bahwere P, Sadler K, Hallam A. Management of severe acute malnutrition in children. Lancet 2006; 368: 1992-2000.
8 Amadi B, Imasiku M, Sakala M, Banda R, Kelly P. Integration of HIV care into community management of acute childhood malnutrition permits good outcomes: retrospective analysis of three years of a programme in Lusaka. PLoS One 2016; 11: e0149218.
9 WHO Guideline: Updates on the management of severe acute malnutrition in infants and children. Geneva: World Health Organization; 2013.
10 Isanaka S, Langendorf C, Berthé F, Gnegne S, Li N, Ousmane N, Harouna S, Hassane H, Schaefer M, Adehossi E, Grais RF. Routine Amoxicillin for Uncomplicated Severe Acute Malnutrition in Children. N Engl J Med 2016; 374: 444-53.
11 Amadi BC, Kelly P, Mwiya M, Mulwazi E, Sianongo S, Changwe F, Thomson M, Hachungula J, Watuka A, Walker-Smith JA, Chintu C. Intestinal and systemic infection, HIV and mortality in Zambian children with persistent diarrhoea and malnutrition. J Ped Gastroenterol Nutr 2001; 32: 550-554.
12 Prendergast A, Kelly P. Interactions between intestinal pathogens, enteropathy and malnutrition in developing countries. Curr Op Infect Dis 2015; 29; 229-236.
13 Trehan I, Kelly P, Shaikh N, Manary MJ. New insights into Environmental Enteric Dysfunction. Arch Dis Child 2016; 101:741-4.
14 Picot J, et al. The effectiveness of interventions to treat severe acute malnutrition in young children: a systematic review. Health Tech Assessment 2012; 16: 19.
15 Kotloff KL, et al. Burden and aetiology of diarrhoeal disease in infants and young children in developing countries (the Global Enteric Multicenter Study, GEMS): a prospective, case-control study. Lancet 2013; 382: 209–22.

16 Platts-Mills JA, et al. Pathogen-specific burdens of community diarrhoea in developing countries: a multisite birth cohort study (MAL-ED). Lancet Glob Health 2015; 3: e564–75.
17 Scrimshaw, NS, et al. Monogr Ser World Health Organ, 1968. 57: 3-329.
18 Mata LJ. The Children of Santa Maria Cauqué: A Prospective Field Study of Health and Growth (International nutrition policy series) MIT Press: 1978.
19 Amadi B, Besa E, Zyambo K, Kaonga P, Louis-Auguste J, Chandwe K, Tarr PI, Denno DM, Nataro JP, Faubion W, Sailer A, Yeruva S, Brantner T, Murray JA, Prendergast AJ, Turner JR, Kelly P. Impaired barrier function and autoantibody generation in malnutrition enteropathy in Zambia. EBiomedicine 2017; 22: 191-199.
20 Kelly P, Besa E, Zyambo K, Louis-Auguste J, Lees J, Banda T, Soko R, Banda R, Amadi B, Watson A. Endomicroscopic and transcriptomic analysis of impaired barrier function and malabsorption in environmental enteropathy. PLoS Negl Trop Dis 2016; 10: e0004600.
21 Trehan I, Goldbach HS, LaGrone LN, Meuli GJ, Wang RJ, Maleta KM, Manary MJ. Antibiotics as part of the management of Severe Acute Malnutrition. N Engl J Med 2013; 368: 425-35.
22 Berkley JA, Ngari M, Thitiri J, Mwalekwa L, Timbwa M, Hamid F, Ali R, Shangala J, Mturi N, Jones KD, Alphan H, Mutai B, Bandika V, Hemed T, Awuondo K, Morpeth S, Kariuki S, Fegan G. Daily co-trimoxazole prophylaxis to prevent mortality in children with complicated severe acute malnutrition: a multicentre, double-blind, randomised placebo-controlled trial. Lancet Glob Health. 2016; 4: e464-73.
23 Attia S, Versloot CJ, Voskuijl W, van Vliet SJ, Di Giovanni V, Zhang L, Richardson S, Bourdon C, Netea MG, Berkley JA, van Rheenen PF, Bandsma RH. Mortality in children with complicated severe acute malnutrition is related to intestinal and systemic inflammation: an observational cohort study. Am J Clin Nutr 2016; 104:1441–9.
24 Marchbank T, Davison G, Oakes JR, Ghatei MA, Patterson M, Moyer MP, Playford RJ. The nutriceutical bovine colostrum truncates the increase in gut permeability caused by heavy exercise in athletes. Am J Physiol Gastrointest Liver Physiol 2011; 300: G477-84.
25 Bode L. The functional biology of human milk oligosaccharides. Early Hum Dev 2015; 91: 619-22.
26 Bode L, Salvestrini C, Park PW, Li JP, Esko JD, Yamaguchi Y, Murch S, Freeze HH. Heparan sulfate and syndecan-1 are essential in maintaining murine and human intestinal epithelial barrier function. J Clin Invest 2008; 118: 229-238.
27 Salvatore S, Heuschkel R, Tomlin S, Davies SE, Edwards S, Walker-Smith JA, French I, Murch SH. A pilot study of N-acetyl glucosamine, a nutritional substrate for glycosaminoglycan synthesis, in paediatric chronic inflammatory bowel disease. Aliment Pharmacol Ther 2000; 14: 1567-1579.
28 Jeppesen PB, Pertkiewicz M, Messing B, Iyer K, Seidner DL, O'Keefe SJ, Forbes A, Heinze H, Joelsson B. Teduglutide reduces need for parenteral support among patients with short bowel syndrome with intestinal failure. Gastroenterol 2012; 143: 1473-1481.
29 Veitch AM, Kelly P, Zulu I, Segal I, Farthing MJG. Tropical enteropathy: a T cell mediated crypt hyperplastic enteropathy. Eur J Gastroenterol Hepatol 2001;13:1175-81.

30 Jones KD, Hünten-Kirsch B, Laving AM, Munyi CW, Ngari M, Mikusa J, Mulongo MM, Odera D, Nassir HS, Timbwa M, Owino M, Fegan G, Murch SH, Sullivan PB, Warner JO, Berkley JA. Mesalazine in the initial management of severely acutely malnourished children with environmental enteric dysfunction: a pilot randomized controlled trial. BMC Med. 2014;12:133.
31 Carter BA, Cohran VC, Cole CR, Corkins MR, Dimmitt RA, Duggan C, Hill S, Horslen S, Lim JD, Mercer DF, Merritt RJ, Nichol PF, Sigurdsson L, Teitelbaum DH, Thompson J, Vanderpool C, Vaughan JF, Li B, Youssef NN, Venick RS, Kocoshis SA. Outcomes from a 12-Week, Open-Label, Multicenter Clinical Trial of Teduglutide in Pediatric Short Bowel Syndrome. J Pediatr. 2017 Feb;181:102-111.e5. doi: 10.1016/j.jpeds.2016.10.027.
32 American Society for Gastrointestinal Endoscopy. Modifications in endoscopic practice for pediatric patients. Gastrointestinal endoscopy 2008; 67: 1-9. doi:10.1016/j.gie.2007.07.008.
33 Thomas JE, Dale A, Bunn JE, Harding M, Coward WA, Cole TJ, Weaver LT. Early *Helicobacter pylori* colonisation: the association with growth faltering in The Gambia. Arch Dis Child. 2004; 89: 1149-54.
34 Kosek M, Haque R, Lima A, Babji S, Shrestha S, Qureshi S, Amidou S, Mduma E, Lee G, Yori PP, Guerrant RL, Bhutta Z, Mason C, Kang G, Kabir M, Amour C, Bessong P, Turab A, Seidman J, Olortegui MP,
Quetz J, Lang D, Gratz J, Miller M, Gottlieb M. Fecal markers of intestinal inflammation and permeability associated with the subsequent acquisition of linear growth deficits in infants. Am J Trop Med Hyg 2013; 88: 390-396.
35 Zulu J, Mwanza-Lisulo M, Besa E, Kaonga P, Chisenga CC, Chomba M, Simuyandi M, Banda R, Kelly P. Improving validity of informed consent for biomedical research in Zambia using a laboratory exposure intervention. PLOS One 2014; 9: e108305.
36 Karrison TG et al. Design of phase II cancer trials using a continuous endpoint of change in tumor size: application to a study of sorafenib and erlotinib in non small-cell lung cancer. J Natl Cancer Inst 2007; 99: 1455-61.
37 Wason JMS, Seaman SR. Using continuous data on tumour measurements to improve inference in phase II cancer studies. Statistics in Medicine 2013; 32: 4639-4650.
38 Moher D, et al. CONSORT 2010 explanation and elaboration: Updated guidelines for reporting parallel group randomised trials. Int J Surgery 2012; 10: 28-55.
39 Wason JM, Stecher L, Mander AP. Correcting for multiple-testing in multi-arm trials: is it necessary and is it done? Trials 2014; 15: 364.

PROTOCOL AMENDMENTS – SUMMARY OF CHANGES

Summary of Changes from version 2.0

Major

1. We have relaxed the inclusion criteria, as we no longer feel we need to wait until a child has transitioned from F75 to F100. This is because is seems to slow up recruitment. Having reached 70 randomisations with very few clinical adverse events we feel we can leave the decision when to randomize to the study clinician. We have also removed this criterion from CRF2 Screening Form.
2. We have made changes to the lab analyses. We would like to increase the number of biomarkers we are testing on the plasma samples, so that we can better see the effect of the treatment on both the inflammatory response, and markers of endothelial activation which are affected by inflammation. This will help with our understanding of the role the gut plays in driving inflammation in these children. The Luminex multiplex platform that analyses all these analytes is a highly specialized machine, and is located in Harare. This does mean that Zambian samples will need to be shipped to Harare to be analysed.
   To reflect this we have made the following changes to the protocol:
   - Added an exploratory endpoint to the study
   - Added the biomarkers in table 11.5 Objectives and Endpoints
   - Added the biomarkers to 13.8.3 Trial assessments
   - Added a line in 14.1 laboratories confirming multiplex samples will be analyzed in Harare.

Minor

1. We have also made minor edits to CRF15 the Serious Adverse Event Reporting form.
   - Added section (e) under 15-2.2 and 15-5.4
   - We have added concomitant medicines in 15-2.7 – there are slots for 10 meds.
   - We have redesigned 15-5.4 so that it adjudicates each event – and no longer do we give an overall summary.
   - This means that 15-5.6 has been removed as it is now included in the 15-5.4.

- We also added 15-5.9 and 15-5.10 connecting the concomitant meds to the adverse event.
- Changed numbers of questions so that it all now flows consecutively
- Added DAIDS grade 5 (death) to the selection options in 15-2.3 and in 15-4.4.

2. We added MCAZ and ZAMRA to the list of IRB’s to report SAE’s to. Page 48

TAME SAP
Statistical Analysis Plan

Administrative Information

Clinical trial information	
Title	Therapeutic approaches to malnutrition enteropathy: phase II trials of four novel interventions in children in Zambia and Zimbabwe
Trial registration number	NCT03716115
SAP version number	3.0
Date of SAP version	12th March 2021
Trial protocol version used to develop SAP	V 3.0, 040121
SAP revisions	None at present time
Roles and responsibility	Dr Kelley VanBuskirk, Trial Statistician<br>Ms Miyoba Chipunza, Trial Coordinator<br>Professor Paul Kelly, Chief Investigator
Signatures	Dr Kelley VanBuskirk<br><br>Ms Miyoba Chipunza<br><br>Professor Paul Kelly

Study Methods

Trial design

Randomized controlled trial, single blinded

Allocation ratio will be 1:1:1:1:1 to the following treatments: colostrum, GlcNAc, teduglutide, budesonide or standard care.

Randomisation

Type: block randomisation stratified by site.

Randomisations will be performed with computer-generated lists created with the ralloc command from the sxd1_4 package in Stata. Each treatment allocation will be printed on a slip and placed in an opaque envelope that will be additionally sealed with tape to prevent unintended unsealing in dry season conditions.

The block size(s) has/have been selected by the trial statistician and will not be disclosed to other members of the study team until the end of the trial.

Sample size

125 children with SAM

Sample size was adjusted downwards in protocol v3.0 to reflect lower than anticipated withdrawals and death:

There are no available data from our represented population to draw upon for an understanding of the baseline variance of the EE biomarker score. The primary outcome response from each patient is the EE biomarker score at 14 days (window 14-18 days) after treatment initiation, adjusted for baseline values. We have assumed that the EE biomarker score is normally distributed with a common standard deviation. To determine the sample size, we drew upon two methods to establish an effect size. We assumed the difference would be larger than the likely inherent imprecision in the measurements of each outcome, and we have defined the magnitude of the effect on a standardised scale. The trial will detect a medium/large effect of Cohen's d effect size of 0.3, with 80% power and 90% confidence, and a conservative correlation between baseline and follow-up estimate of 0.5, we will need a sample size of 23 per group across 5 groups to analyse with the ANCOVA method. We expect there to be approximately 5% loss to follow up due to deaths another reasons, such as drug intolerance or withdrawal. Adjusting our

sample size of 115 for 5% loss to follow-up and rounding up to a multiple of 5, we aim to randomise 125 patients in total (25 in each group). We will evaluate our withdrawal rate again and adjust the sample size accordingly towards the end of the trial.

Framework

Superiority to current standard care practices.

Statistical interim analyses and monitoring guidelines

Interim analyses for treatment efficacy will not be performed. Monitoring of adverse events will take place throughout the study and SAEs or SUSARs will be reviewed every 6 months by the DMEC, with an emphasis on searching for trends suggesting unexpected adverse events.

Stopping guidance

The decision to prematurely terminate an arm of the trial due to SAEs or SUSARs will depend on their severity and the total count of SAEs/SUSARs. If an unequivocal excess morbidity or mortality is observed in a treatment arm, that arm of the study will be discontinued. Excess morbidity and mortality will be defined
based on 1) standard SAM mortality rates observed in the study hospitals and 2) morbidity and mortality events in the control arm. Any discontinuation decision would be based on the clinical and ethical judgment of the DMEC.

Timing of final analysis

Databases will be finalised and final analysis will be initiated within 7-14 days after measurement of primary and secondary endpoints is complete.

Timing of outcome assessments

See trial protocol for timing of different outcome assessments.

Statistical Principles

Confidence intervals and p-values

An alpha of 0.1 will be utilised for the all analyses. We will report 90% confidence intervals.

We will not undertake any adjustments of the false positive (type I) error rate, since the aim of this trial is to inform the treatment development process.

Adherence and protocol deviations

We have allowed for 5% loss due to death and withdrawal. Once withdrawn, lost participants will not be replaced. Data on protocol deviations will be collected, including the child's study ID, nature of the deviation, date and time of occurrence, precipitating circumstances, and relevant follow-up observations. The commonest anticipated protocol deviation will be doses of IMP withheld due to patient ill-health: vomiting, diarrhea, etc. Analysis will include adjustment for whether a child received 80% or more of planned doses or not. Doses count as correctly administered if given within 2 hours of the scheduled time.

Analysis populations

Analysis sets will include the intention-to-treat and per-protocol populations. The main analysis will be performed on the full trial population according to the per- protocol principle, as the study's intent is evaluation of candidates for a Phase 3 trial.

Data from the subgroup of children receiving endoscopy will be additionally analysed separately.

Trial population

Methodological details on screening, eligibility, recruitment, withdrawal, and follow-up may be found in the trial protocol.

Baseline patient characteristics

We will report medians, ranges and interquartile ranges for age, WAZ and HAZ. Biological sex will be reported as percentages. These will be calculated and reported both by site and as combined totals for the trial population.

Analysis

Outcome definitions
These are described in detail in the trial protocol.

Analysis methods

Comparisons of the environmental enteropathy activity score and secondary endpoints in each treatment group versus the control group will be performed using mixed effects ANCOVA models. For both primary and secondary outcomes, we will adjust for a core set of baseline variables. These are: sex (male/female); oedema (yes/no); HIV status (yes/no); diarrhoea (yes/no); baseline WLZ scores (continuous); baseline biomarker/histology scores (continuous); and site. Study site is the stratification variable. Through due diligence, the TAME trial team aim to have complete capture of all data from all patients. If mixed effects ANCOVA generates unstable models due to reduced sample size, the trial statistician will confer with statisticians on the TSC and DMEC to identify alternative approaches.

Missing or spurious data

No attempt will be made to account for missing data. Implausible values will be corrected while the trial participant is on the ward, or will be dropped.

Deviations from the Statistical Analysis Plan

Any changes from the original analysis plan will be discussed by the Trial Management Group. Deviations from the plan will be reported to the Sponsor, Trial Steering Committee, and Data Management and Ethics Committee.

Additional analyses

Harms

SAEs of grade 3/4 will be analysed. Deaths will be tabulated separately.

Statistical software

Primarily we will use Stata v. 15; R version 3.4.0 and R Studio v. 1.0.143 will be employed as needed.

References

TAME Trial Protocol v0.8_100518.

TAME Data Management Plan.

TAME Trial Master File.

TAME Statistical Master File.

Health Research Act 2013, Zambia.

Medicines and Allied Substances Act 2013, Zambia.

The Medicines and Allied Substances Control Act (MASCA) Chapter 15:03 Part III, Medicines Control Authority of Zimbabwe.

The Medicines and Allied Substances Control Regulations (MASCR) SI 150 (1991), Medicines Control Authority of Zimbabwe.

Wason JM, Stecher L, Mander AP. Correcting for multiple-testing in multi-arm trials: is it necessary and is it done? Trials 2014; 15: 364.
