# Supplementary material for: Malnutrition enteropathy in Zambian and Zimbabwean children with severe acute malnutrition: A multi-arm randomized phase II trial
Source: Nat Commun. 2024 Apr 17;15:2910. doi: 10.1038/s41467-024-45528-0 (PMC11024201; doi:10.1038/s41467-024-45528-0)
Supplement: Supplementary file 3 — Reporting Summary [file 41467_2024_45528_MOESM3_ESM.pdf]

nature portfolio

Corresponding author(s): Paul Kelly

Last updated by author(s): Dec 14, 2023

Reporting Summary

Nature Portfolio wishes to improve the reproducibility of the work that we publish. This form provides structure for consistency and transparency in reporting. For further information on Nature Portfolio policies, see our Editorial Policies and the Editorial Policy Checklist.

Statistics

For all statistical analyses, confirm that the following items are present in the figure legend, table legend, main text, or Methods section.

n/a	Confirmed	
☐	☒	The exact sample size (*n*) for each experimental group/condition, given as a discrete number and unit of measurement
☐	☒	A statement on whether measurements were taken from distinct samples or whether the same sample was measured repeatedly
☐	☒	The statistical test(s) used AND whether they are one- or two-sided<br>*Only common tests should be described solely by name; describe more complex techniques in the Methods section.*
☐	☒	A description of all covariates tested
☐	☒	A description of any assumptions or corrections, such as tests of normality and adjustment for multiple comparisons
☐	☒	A full description of the statistical parameters including central tendency (e.g. means) or other basic estimates (e.g. regression coefficient) AND variation (e.g. standard deviation) or associated estimates of uncertainty (e.g. confidence intervals)
☐	☒	For null hypothesis testing, the test statistic (e.g. *F*, *t*, *r*) with confidence intervals, effect sizes, degrees of freedom and *P* value noted<br>*Give P values as exact values whenever suitable.*
☒	☐	For Bayesian analysis, information on the choice of priors and Markov chain Monte Carlo settings
☒	☐	For hierarchical and complex designs, identification of the appropriate level for tests and full reporting of outcomes
☐	☒	Estimates of effect sizes (e.g. Cohen's *d*, Pearson's *r*), indicating how they were calculated

Our web collection on statistics for biologists contains articles on many of the points above.

Software and code

Policy information about availability of computer code

Data collection	No new code used
Data analysis	Data were analysed using Stata 15 and Stata 17

For manuscripts utilizing custom algorithms or software that are central to the research but not yet described in published literature, software must be made available to editors and reviewers. We strongly encourage code deposition in a community repository (e.g. GitHub). See the Nature Portfolio guidelines for submitting code & software for further information.

Data

Policy information about availability of data

All manuscripts must include a data availability statement. This statement should provide the following information, where applicable:

- Accession codes, unique identifiers, or web links for publicly available datasets
- A description of any restrictions on data availability
- For clinical datasets or third party data, please ensure that the statement adheres to our policy

Data supporting the findings of this study are available in the article and in its Supplementary Information. Source Data are provided as a Source Data file for Figure 3, and source data have been deposited in Figshare under accession code https://doi.org/10.6084/m9.figshare.24442699. The data uploaded to Figshare include deidentified individual participant data, trial protocol, and statistical analysis plan, and all data will be freely available from the Nature Communications website from the date of publication. Source data for Figure 3 are provided with this paper and have been uploaded to Figshare.

Research involving human participants, their data, or biological material

Policy information about studies with human participants or human data. See also policy information about sex, gender (identity/presentation), and sexual orientation and race, ethnicity and racism.

Reporting on sex and gender	Both sexes were included and data on sex have been reported in Table 1
Reporting on race, ethnicity, or other socially relevant groupings	All children were indigenous African.
Population characteristics	Children admitted to two African hospitals, in Lusaka (Zambia) and Harare (Zimbabwe) with Severe Acute Malnutrition
Recruitment	Children were recruited consecutively by approaching mothers on the wards. As can be seen from Figure 1 (CONSORT flowchart) the number of children ineligible was small so the risk of bias in recruitment was low.
Ethics oversight	Ethics approval was obtained from the University of Zambia Biomedical Research Ethics Committee (006-09-17, dated 9th July 2018), the National Health Research Committee of Zambia, the Zambia Medicines Regulatory Authority (CT 082/ 18), the Joint Research Ethics Committee of Harare Central Hospital (JREC/66/19), The Medicines Control Authority of Zimbabwe (CT/176/2019), and the Medical Research Council of Zimbabwe (MRCZ/A/2458).

Note that full information on the approval of the study protocol must also be provided in the manuscript.

Field-specific reporting

Please select the one below that is the best fit for your research. If you are not sure, read the appropriate sections before making your selection.

☒ Life sciences ☐ Behavioural & social sciences ☐ Ecological, evolutionary & environmental sciences

For a reference copy of the document with all sections, see nature.com/documents/nr-reporting-summary-flat.pdf

Life sciences study design

All studies must disclose on these points even when the disclosure is negative.

Sample size	The original sample size for this phase II trial was 225 children (45 in each of the five arms). This was based on the composite stool biomarker score, derived from Kosek et al (2013) as previously described. Enrolment was slower than anticipated due to COVID-19, but trial losses due to deaths, drug intolerance or withdrawal were also substantially lower than anticipated (3% observed against 20% anticipated). The Trial Steering Committee and DMEC therefore reviewed the sample size in January 2021, once 82 children had been enrolled, and the decision was made to reduce the planned sample size. The new sample size was based on a medium/large Cohen's d effect size of 0.3, with 80% power and 90% confidence, and a conservative correlation between baseline and follow-up estimate of 0.5, requiring a sample size of 23 per group across 5 groups to analyse with the ANCOVA method. Allowing for 5% losses, the sample size of 115 was rounded up to 125 participants in total (25 in each group).
Data exclusions	No data were excluded
Replication	Primary endpoint ELISAs were run in duplicate and very high concordance (r^2 0.98) were observed
Randomization	Trial identification numbers were allocated sequentially in each site and randomisation was carried out by opening a sealed envelope bearing the corresponding number. The randomisation sequence was prepared by the trial statistician (KVB), stratified by study site, in random permuted blocks of variable size.
Blinding	Because this was a multi-arm trial, full blinding would have required placebos for each intervention, so clinical staff were not blinded. However, laboratory staff were blinded to treatment allocation.

Reporting for specific materials, systems and methods

We require information from authors about some types of materials, experimental systems and methods used in many studies. Here, indicate whether each material, system or method listed is relevant to your study. If you are not sure if a list item applies to your research, read the appropriate section before selecting a response.

Materials & experimental systems

n/a	Involved in the study
☒	☐ Antibodies
☒	☐ Eukaryotic cell lines
☒	☐ Palaeontology and archaeology
☒	☐ Animals and other organisms
☐	☒ Clinical data
☒	☐ Dual use research of concern
☒	☐ Plants

Methods

n/a	Involved in the study
☒	☐ ChIP-seq
☒	☐ Flow cytometry
☒	☐ MRI-based neuroimaging

Clinical data

Policy information about clinical studies

All manuscripts should comply with the ICMJE guidelines for publication of clinical research and a completed CONSORT checklist must be included with all submissions.

Clinical trial registration: The trial was registered at www.clinicaltrials.gov as NCT03716115

Study protocol: The protocol has been uploaded to Figshare, and a trial protocol paper was published in BMJ Open.

Data collection: The TAME trial was conducted in the University Teaching Hospital, Lusaka, and Harare Central Hospital, Harare. Patients were recruited and followed up from 4th May 2020 to 25th May 2021.

Outcomes: The primary endpoint was composite faecal inflammatory biomarker score, comprising myeloperoxidase, neopterin and alpha1-antitrypsin, all measured in samples taken on day 15 by ELISA by laboratory scientists blinded to study arm. Secondary endpoints were evaluated at day 15: changes in anthropometry (weight, weight-for-height, and MUAC), plasma concentrations of biomarkers of enteropathy, microbial translocation and systemic inflammation (iFABP, LBP, CRP, sCD14 and GLP-2), days with diarrhoea, fever, or oedema, and adverse events (see below). Secondary endpoints also included mortality between recruitment and day 28. Analysis of oedema was restricted to those with oedema at baseline.

Plants

Seed stocks: n/a

Novel plant genotypes: n/a

Authentication: n/a
